# Mammalian cell-derived extracellular vesicles remodel the immune-repair microenvironment in osteoarthritis: from pathological signal transmission to regenerative therapy

**DOI:** 10.3389/fimmu.2026.1936617

**Published:** 2026-09-03

**Authors:** Lin Zhang, Xuxu Yang, Yang Yu, Lidan Yang

**Affiliations:** Department of Orthopedics, Affiliated Hospital of Zunyi Medical University, Zunyi, China

**Keywords:** cartilage regeneration, exosomes, extracellular vesicles, immune microenvironment, osteoarthritis

## Abstract

Osteoarthritis is a whole-joint disease involving articular cartilage, synovium, subchondral bone, fat pad, meniscus, blood vessels, and sensory nerves. Its core pathology is not simply passive wear of the cartilage matrix, but rather reciprocal driving forces among mechanical stress, low-grade inflammation, cellular senescence, immunometabolic reprogramming, and failure of endogenous repair. As lipid bilayer-enclosed intercellular information carriers, Extracellular vesicles (EVs) carry proteins, lipids, nucleic acids, and metabolites, establishing a communication network among joint tissues that is source-dependent and context-dependent. In the osteoarthritic state, EVs released by inflammatory synovial fibroblasts, macrophages, stressed chondrocytes, osteocytes, and vascular endothelial cells propagate pro-inflammatory, catabolic, senescence, and mitochondrial damage signals, driving synovitis, cartilage matrix degradation, abnormal subchondral bone remodeling, and vascular invasion, thus forming a self-sustaining pathological EV cycle. Conversely, EVs derived from mesenchymal stromal cells (MSCs), regulatory macrophages, and other therapeutic cells can curb excessive inflammation, modulate macrophage status, restore chondrocyte autophagy and mitochondrial quality control, attenuate apoptosis, ferroptosis, and senescence, and translate immunosuppression into regenerative initiation signals by re-establishing extracellular matrix metabolism, recruiting endogenous repair cells, and coordinating osteochondral interface remodeling. In recent years, donor cell preconditioning, nucleic acid and protein cargo editing, cartilage-targeted modification, and hydrogel sustained-release systems have further improved the efficacy, tissue selectivity, and intra-articular retention of EVs. However, EV heterogeneity, inconsistent nomenclature and isolation methods, non-uniform dosage units, lack of potency assays, and insufficient long-term safety evidence still limit clinical translation. Focusing on the main thread of “pathological EV cycle — immune resetting — regenerative initiation”, this review systematically elaborates the dual roles of EVs in the osteoarthritis immune-repair ecosystem and discusses key issues from mechanistic research and engineering design to clinical-grade manufacturing, with the aim of providing a new theoretical framework for disease-modifying therapy of osteoarthritis.

## 1 Introduction

Osteoarthritis is one of the leading musculoskeletal diseases causing chronic pain, activity limitation, and functional disability in middle-aged and elderly populations (1, 2). Traditional views often describe it as cartilage “wear and tear” caused by aging and mechanical loading. However, this explanation cannot fully account for early synovitis, discordance between pain and radiographic damage, rapid progression of post-traumatic osteoarthritis, and marked differences in disease course among patients. Current research has redefined osteoarthritis as a whole-joint disease driven jointly by mechanical abnormalities, immune inflammation, metabolic disorders, cellular senescence, and repair failure (3, 4). Matrix fragments released from articular cartilage can activate synovial cells and innate immune responses. Activated synovium then produces cytokines, chemokines, and proteases, which further promote cartilage degradation. This establishes a positive feedback loop between synovitis and cartilage damage (5). Subchondral bone remodeling, angiogenesis, adipose tissue inflammation, and sensory nerve sensitization extend this local feedback into a cross-tissue pathological network (6). Studies have pointed out that a continuously amplifying feedback relationship exists between cartilage degradation products and synovial inflammation (7). This understanding provides a pathological basis for understanding EV-mediated cross-tissue communication.

EVs are membrane-bound particles actively released by cells. They can protect and deliver proteins, lipids, messenger RNA (mRNA), microRNA (miRNA), long non-coding RNA (lncRNA), circular RNA (circRNA), and metabolites in the extracellular environment (8). Based on their biogenesis, they are traditionally classified into exosomes derived from intraluminal EVs of multivesicular bodies, microEVs formed by direct budding from the plasma membrane, and apoptotic EVs generated during apoptosis (9). EVs are particularly suitable for explaining the complexity of osteoarthritis. They are not simple substitutes for single soluble factors. Instead, they are combined information carriers that simultaneously carry multiple classes of bioactive molecules. A single EV may simultaneously affect inflammatory receptors, transcription factors, metabolic enzymes, and cell death pathways. Different EV subpopulations can have completely different or even opposite effects depending on source tissue, cell state, mechanical load, oxygen tension, and inflammatory stimuli (10). EVs in the pathological joint can act as mediators of inflammation and tissue damage. In contrast, EVs released by therapeutic cells can suppress excessive immune responses and shift recipient cells from stress and catabolic states toward survival, synthesis, and repair. Therefore, EVs should not be broadly viewed as natural anti-inflammatory nanoparticles. They are better understood as “state transition instructions” within the joint immune-repair ecosystem.

Previous reviews have primarily organized therapeutic EVs evidence around mesenchymal stromal cell-derived exosomes, exosomal miRNAs, or cartilage/chondrocyte repair themes (11–13). These frameworks have provided an important foundation for understanding the therapeutic potential of EVs. However, an organization centered on source cells or single cargo classes may relatively weaken the bidirectional and multidirectional EV communication among synovium, cartilage, subchondral bone, immune cells, and other joint tissues. In fact, whether therapeutic EVs can truly modify disease progression depends on whether they can simultaneously address three interrelated issues. First, they must interrupt the pathological EV cycle maintained jointly by synovium, cartilage, bone, and adipose tissue. Second, they must reset the pro-inflammatory, immune-senescent, and metabolically abnormal joint environment to a state that permits repair. Third, they must convert short-term anti-inflammatory effects into sustained matrix reconstruction and osteochondral unit repair. Based on this understanding, this review follows the main thread of “pathological EV cycle – immune reset – regeneration initiation”. It explores how EVs transform from inflammatory messengers into regenerative instructions. It also analyzes the mechanistic, manufacturing, and evaluation issues that need to be addressed for their development into clinical-grade biologics.

## 2 From local cartilage injury to whole-joint communication disorder: EVs-mediated osteoarthritis immune-repair ecosystem

### 2.1 Osteoarthritis as a disorder of whole-joint immune, mechanical, and metabolic homeostasis

A healthy joint is not simply formed by independent tissues pieced together. Cartilage bears and distributes loads. Synovial fluid provides lubrication and nutrition. The synovium maintains fluid homeostasis and participates in immune surveillance. Subchondral bone regulates mechanical transduction. The adipose tissue, menisci, ligaments, blood vessels, and nerves jointly participate in stress perception and injury repair (14). Chondrocytes reside in a hypoxic, avascular, and high mechanical load environment. Their homeostasis depends heavily on autophagy, mitochondrial quality control, extracellular matrix feedback, and paracrine signals released by adjacent tissues (15). When joint instability, obesity, aging, or trauma disrupts this balance, mechanosensitive pathways, oxidative stress, DNA damage, and innate immune signals can be synchronously activated. This shifts chondrocytes from a quiescent matrix-maintaining phenotype toward an inflammatory, hypertrophic-like, or senescent phenotype (16). The synovium is not only a site of inflammation in this process. It is also an interface where signals from different tissues converge and are redistributed. After synovial macrophages recognize cartilage matrix fragments, crystals, and damage-associated molecular patterns (DAMPs), they can release interleukin-1β (IL-1β), tumor necrosis factor-α (TNF-α), interleukin-6 (IL-6), chemokines, and lipid mediators. Synovial fibroblasts can then produce matrix metalloproteinases (MMPs), ADAMTS family proteases, and molecules that promote immune cell recruitment (17–19). Meanwhile, the subchondral bone may show increased bone turnover and trabecular structural changes in early disease, followed by sclerosis, osteophyte formation, and abnormal vascular invasion.

These changes do not occur in parallel. Instead, they continuously exchange information through soluble molecules and EVs in the synovial fluid. Therefore, the “lesion” in osteoarthritis is more like a communication network composed of multiple tissue nodes that continually renews itself. Immune responses in this network have distinct phases. Moderate inflammation after acute injury helps clear necrotic cells and matrix debris and promotes vascular and repair cell recruitment. However, if inflammation cannot be terminated in time, persistent macrophage activation, fibroblast responses, and cellular senescence will convert programs originally serving repair into chronic tissue destruction (20). Thus, the therapeutic goal cannot stop at lowering a single inflammatory cytokine. Instead, it should restore the temporal sequence of inflammation initiation, damage clearance, immune resolution, and tissue reconstruction. EVs are precisely at the center of this transition process. They can transmit signals that “continue inflammation” as well as instructions that “stop damage and begin repair” (21, 22).

### 2.2 Formation, heterogeneity, and biological information encoding of EVs

The formation of EVs is not random leakage of cellular contents. In the endosomal pathway, plasma membrane proteins and cytosolic components are internalized into early endosomes. Subsequently, with the involvement of the endosomal sorting complexes required for transport (ESCRT), tetraspanins, lipid rafts, and ceramide-related mechanisms, multivesicular bodies form. After multivesicular bodies fuse with the plasma membrane, the small EVs within them are released. EVs formed by direct budding from the plasma membrane are regulated by membrane lipid asymmetry, actin rearrangement, calcium signaling, and small GTPases (23–25). EVs formed during apoptosis may also carry organelles, nucleic acid fragments, and “eat-me” signals. Particles generated by different biogenesis pathways overlap in size. Therefore, particle size alone cannot prove their origin. More importantly, the cargo of EVs is subject to selective sorting. RNA-binding proteins can recognize specific RNA sequences or modification states. This causes certain miRNAs, lncRNAs, or mRNAs to be enriched in EVs, while others are retained within the cell (26). Inflammation, hypoxia, mechanical stress, and metabolic alterations in the osteoarthritic environment can modulate this sorting process. After reaching recipient cells, EVs can enter cells through receptor-ligand binding, clathrin- or caveolin-mediated endocytosis, macropinocytosis, phagocytosis, and membrane fusion. Their surface integrins, tetraspanins, glycans, and phospholipid composition affect tissue homing. The inflammatory state, matrix environment, and phagocytic capacity of recipient cells in turn determine uptake efficiency (27). Synovial macrophages have strong particle uptake capacity and may be the primary recipients of EVs injected intra-articularly. Chondrocytes are embedded in dense, negatively charged cartilage extracellular matrix. Whether natural EVs can enter deep cartilage is limited by size, surface charge, binding proteins, and cartilage integrity (28). This means that efficient uptake observed in monolayer cultures *in vitro* cannot directly represent delivery to deep cartilage *in vivo*.

Furthermore, EV secretion should not be viewed solely as a mechanism for intercellular information exchange. Their release can also be an important pathway for cells to eliminate unwanted, damaged, or potentially toxic substances. For example, Takahashi et al. demonstrated that when exosome release is inhibited, cytosolic nuclear DNA accumulates. This subsequently triggers DNA damage responses and innate immune signaling and disrupts cellular homeostasis. Exosome secretion helps cells expel harmful cytosolic DNA (29). Therefore, not all cargo in EVs should be automatically interpreted as “biological information” purposefully selected and encoded by donor cells. Some cargo may primarily reflect the cell’s own quality control and waste clearance processes. However, these molecules, once released, may still exert biological effects on recipient cells. This point is particularly important for osteoarthritis. Damaged or unwanted molecular components expelled by stressed or senescent chondrocytes to maintain their own homeostasis, once taken up by surrounding cells, may still be converted into signals with pathological significance.

The biological effects of EVs should also not be reduced to a single miRNA-target gene axis. A EV preparation simultaneously contains multiple RNAs, proteins, lipids, and metabolites. Their effects may arise from the synergistic regulation of inflammation, cell metabolism, membrane receptors, and organelles by different components. Only under conditions such as removing EVs, disrupting membrane structure, knocking down specific cargo, blocking receptors, or comparing equivalent amounts of free cargo can one more robustly demonstrate the necessity and sufficiency of a particular component for the effect.

### 2.3 Effects of EVs are determined jointly by source cells, cargo composition, recipient cells, and disease context

EVs released by the same cell type in different states can produce opposite effects. Quiescent synovial fibroblasts may participate in matrix homeostasis. However, after stimulation with IL-1β or other inflammatory stimuli, the inflammatory and catabolic signals carried by their EVs are significantly enhanced (21, 30). EVs from mesenchymal stromal cells generally have immunomodulatory and tissue-protective effects. However, donor age, obesity, metabolic disease, tissue source, culture passage, oxygen concentration, medium composition, and three-dimensional culture methods can all alter EV yield and cargo (31–33). Mesenchymal stromal cell EVs obtained from aged or metabolically abnormal donors may lack sufficient mitochondrial protection and anti-inflammatory capacity, or may even carry detrimental signals. Therefore, “mesenchymal stromal cell EVs” is not a fixed drug name. It is a class of complex biological products highly dependent on source and manufacturing process.

Recipient cell state also determines the direction of the response. After inflammatory macrophages uptake EVs, they may undergo glycolysis, lipid metabolism, and epigenetic reprogramming. Chondrocytes under oxidative stress rely more on the regulation of autophagy, mitochondria, and antioxidant systems by EVs (34, 35). The same EVs may mainly suppress synovitis in early inflammatory osteoarthritis. However, in late-stage disease with severe cartilage loss, simply enhancing chondrocyte survival may be insufficient to produce structural repair (15). If the cartilage surface is already highly fibrotic or calcified, tissue penetration of EVs will also be significantly limited. Therefore, when evaluating EV efficacy, recipient cells, disease stage, and joint structural status must be included in the same experimental framework.

Tissue matching effects also exist between source and recipient. Synovial mesenchymal stromal cells have strong tissue relevance to the joint microenvironment. Their EVs may be more favorable for chondrogenesis and synovial immune regulation. Adipose-derived mesenchymal stromal cells are easily accessible and yield high quantities. However, donor obesity and adipose tissue site can affect their secretion profile. Umbilical cord mesenchymal stromal cells have good expansion capacity and lower donor age-related variability. However, they may lack certain tissue-specific signals from adult joints (36, 37). Comparative studies have shown that EVs from bone marrow, adipose, synovium, infrapatellar fat pad, and umbilical cord are not completely consistent in miRNA composition, matrix protective capacity, and immune effects. A comparison between infrapatellar fat pad and subcutaneous adipose-derived EVs found that infrapatellar fat pad mesenchymal stromal cell EVs had advantages in inhibiting matrix degradation. Based on this, miR-99b-3p related to ADAMTS4 regulation was identified. Such studies suggest that tissue source differences themselves may constitute an important basis for therapeutic product screening (37).

### 2.4 EVs form a spatiotemporally selective cross-tissue communication network within the joint

EVs communication within the joint has obvious spatial barriers. The synovium is in direct contact with the synovial fluid. EVs released by inflammatory synovial cells easily enter the joint cavity and are taken up by adjacent macrophages, fibroblasts, and superficial chondrocytes (38). Deep chondrocytes and subchondral bone are separated by calcified cartilage and the tidemark. However, microfissures, vascular invasion, and increased permeability at the osteochondral interface that occur during osteoarthritis can create channels for EVs from subchondral bone to migrate to cartilage (39, 40). Mechanical load also alters interstitial fluid flow and EV transport. This may create EV concentration and cargo environments in weight-bearing regions that differ from non-weight-bearing regions (41). In addition, articular cartilage has distinct spatial zonal structures. Chondrocytes in different zones differ significantly in morphology, mechanical load, oxygen environment, and extracellular matrix composition. It is thus speculated that EV generation, cargo sorting, extracellular retention, and recipient cell accessibility may also have certain zone-dependent characteristics. Direct evidence on this issue has only recently begun to emerge. A recent spatial analysis study of bovine articular cartilage found that EV/CD9-positive structures around chondrocytes in the superficial layer exhibited non-uniform spatial distribution. This distribution pattern changed during early cartilage degeneration (42). However, it has not yet been proven that under normal physiological conditions, chondrocytes in the superficial, middle, and deep zones can stably produce EV subpopulations with clearly different molecular compositions. Future studies could combine spatial sampling and single-cell analysis to determine whether different cartilage zones have unique EV molecular signatures.

The temporal dimension is equally critical. In the early post-traumatic period, EVs may participate in damage sensing, immune cell recruitment, and matrix clearance. After inflammation persists, pathological EVs from macrophages, fibroblasts, and senescent chondrocytes gradually predominate, driving catabolism and aberrant repair. In late disease, reduced chondrocyte numbers, subchondral bone sclerosis, and synovial fibrosis further alter EV source composition and tissue accessibility (43). Collecting synovial fluid only at the endpoint of disease for EV omics analysis makes it difficult to distinguish which changes are drivers and which are merely consequences of severe tissue damage. Kolhe et al. analyzed miRNAs in synovial fluid EVs from osteoarthritis and non-osteoarthritis patients. They found that the EV miRNA profile changed not only with disease but also exhibited sex differences. Female osteoarthritis-related miRNAs were linked to estrogen response and Toll-like receptor (TLR) pathways (44). This study suggests that patient sex, hormonal environment, and disease stage may all affect the EV communication network. It also indicates that a single “osteoarthritis EV signature” may not apply to all patients. Subsequent studies further found altered expression of mRNA, lncRNA, and circRNA in synovial fluid EVs and constructed competing endogenous RNA (ceRNA) regulatory networks. However, such omics associations still need to be confirmed for pathological significance through cell source tracing and functional intervention. Related synovial fluid exosome transcriptome studies provide a basis for EVs as biomarkers for disease stratification and dynamic monitoring (45).

## 3 Pathological EVs as inflammatory messengers: establishment and amplification of the vicious cycle in osteoarthritis

### 3.1 EVs from synovial fibroblasts and inflammatory macrophages amplify synovitis

Synovial fibroblasts function to maintain synovial structure, produce synovial fluid components, and support immune cell survival. After stimulation by cartilage debris, mechanical injury, or inflammatory factors, some fibroblasts transform into a pro-inflammatory and tissue-destructive phenotype. They secrete proteases, chemokines, and EVs. EVs protect these signals from degradation by extracellular nucleases and proteases. They can also diffuse to distant areas within the synovial fluid. This extends focal synovial reactions into an inflammatory environment throughout the joint (46). Kato et al. demonstrated early on that exosomes from IL-1β-stimulated synovial fibroblasts could upregulate MMP13 and ADAMTS5 in normal chondrocytes. At the same time, they downregulated COL2A1 and ACAN and promoted glycosaminoglycan release from cartilage tissue (21). The significance of this study was not limited to finding changes in certain catabolic indicators. It showed that inflammatory fibroblasts could “copy” their own state to chondrocytes through membrane-bound particles, causing the latter to actively participate in matrix destruction. EVs thus become structured information packets connecting synovitis and cartilage degradation, not merely accompanying products in the inflammatory environment. Recent studies have further extended the role of fibroblast EVs from “directly damaging chondrocytes” to “first reprogramming macrophages, then indirectly damaging cartilage.” One study found that exosome release from osteoarthritis synovial fibroblasts increased. These EVs could promote a shift of macrophages toward a pro-inflammatory state and enhance glycolysis. Chondrocytes subsequently treated with the conditioned medium from these macrophages developed an osteoarthritis-like phenotype. Intra-articular injection of such EVs also aggravated synovitis and osteoarthritis progression in mice. Glycolysis inhibition partially attenuated this effect (34). This indicates that pathological EVs not only carry cytokines or miRNAs. They may also increase the persistence of inflammatory responses by reshaping immune cell energy metabolism. Studies on small EVs from human osteoarthritis synovial fibroblasts similarly found that they could regulate chondrocyte inflammation, repair, and catabolic responses. The miRNA composition in the EVs was not simply consistent with that of the parent cells. Related studies emphasize that it is necessary to verify EV effects in disease-relevant primary cells. This is because immortalized cell lines and high-concentration single cytokine stimulation may not reflect the complex state of different fibroblast subpopulations in patient synovium (30). Another study pointed out that synovial fibroblast EVs could induce chondrocyte inflammation through a miR-21-5p/KLF6-related mechanism. This further supports the involvement of synovial EV cargo in disease transmission (47).

Macrophage EVs constitute another major pathway for transmitting synovitis to cartilage. Macrophages present a continuous, plastic spectrum of states at different disease stages, rather than being strictly divided into M1 and M2 categories. EVs released by pro-inflammatory macrophages can carry miRNAs, inflammation-related proteins, and lipids. They reduce chondrocyte autophagy and antioxidant capacity (48, 49). One study found that synovial pro-inflammatory macrophage EVs delivered miR-155-5p to chondrocytes. This promoted cartilage degeneration by inhibiting GSK-3β/mTORC1-related autophagy regulation. Deletion of miR-155 in macrophages alleviated synovitis and cartilage damage in mice. The researchers further constructed engineered adipose-derived mesenchymal stromal cell EVs with pro-inflammatory macrophage affinity peptides. These were used to deliver antagomiR-155-5p. This formed a closed-loop strategy of “identifying pathological immune cells – blocking pathogenic EV cargo – protecting cartilage” (50).

### 3.2 EVs from chondrocytes and senescent cells spread catabolism and senescence phenotypes

Chondrocytes are both recipients of pathological EVs and important sources. In the healthy state, chondrocyte EVs may participate in matrix mineralization control, intercellular homeostasis, and chondrogenesis. Under conditions of inflammation, abnormal shear stress, oxidative stress, or senescence, their EV cargo changes. Stressed chondrocytes can transmit catabolic and senescence-related signals to neighboring chondrocytes, synovial cells, or mesenchymal stromal cells through EVs. This allows abnormalities originally confined to a small number of damaged cells to spread to larger areas (51–53). Mao et al. found that miR-95-5p in exosomes from osteoarthritic chondrocytes was lower than in normal chondrocytes. Chondrocyte EVs overexpressing miR-95-5p could target HDAC2 and HDAC8, promoting chondrogenesis and matrix expression. Inhibition of miR-95-5p produced the opposite results (54). This study indicates, on the one hand, that loss of protective cargo in chondrocyte EVs may participate in disease. On the other hand, it shows that pathological EVs may not necessarily act by gaining pro-inflammatory molecules. They may also cause decreased recipient cell function due to the absence of signals needed to maintain homeostasis.

Cellular senescence further alters EV communication. Senescent chondrocytes stop proliferating and form a senescence-associated secretory phenotype (SASP). This SASP is composed of cytokines, chemokines, matrix proteases, lipid mediators, and EVs (55, 56). Its effects are not limited to the senescent cells themselves. It can induce paracrine senescence in neighboring cells, reduce the repair capacity of progenitor cells, and maintain macrophage and fibroblast activation (57). The EV membrane can protect the miRNAs and proteins within, allowing senescence signals to persist longer in the joint cavity. Compared with single soluble SASP factors, senescent EVs may simultaneously alter DNA damage responses, mitochondrial metabolism, and epigenetic states in recipient cells (55, 58). However, current research on “senescent chondrocyte-specific EV cargo” is still less abundant than research on the overall SASP. Many experiments use hydrogen peroxide, IL-1β, or long-term passaging to induce senescence. However, they do not adequately distinguish between EVs released by cellular senescence, acute stress, and apoptosis. Some studies also do not exclude lipoproteins, protein aggregates, and media-derived particles. Therefore, future studies need to combine single-cell transcriptomics, spatial omics, and EV source tracing. This will determine the EV contribution of different chondrocyte subpopulations at various disease stages and verify whether these EVs are sufficient to induce sustained paracrine senescence *in vivo*. Hypoxia-cultured adipose stem cell EVs were shown to alleviate the SASP of osteoarthritic chondrocytes and inhibit disease progression in rats. This supports from the opposite direction that therapeutic EVs can modulate senescence spread (59). Related studies show that therapeutic EVs do not merely make chondrocytes “secrete fewer inflammatory factors.” They may also change the direction of cells from maintaining senescence toward restoring homeostasis (60). Meanwhile, FTO-overexpressing bone marrow mesenchymal stromal cell-derived EVs could inhibit chondrocyte senescence and apoptosis and enhance autophagy (61). This suggests that RNA modification regulation may also become an entry point for reversing the senescent EV network.

### 3.3 EVs from subchondral bone, blood vessels, and adipose tissue drive cross-tissue pathology

Subchondral bone in osteoarthritis is not a passive responder to cartilage wear. Early abnormal bone turnover, osteocyte mechanosensing, bone marrow lesions, and vascular changes can precede obvious cartilage defects (62). Osteocytes reside within the lacunar-canalicular network. They can sense local strain and fluid shear stress. Through soluble factors and EVs, they transmit information to bone surface cells and adjacent cartilage. As calcified cartilage microfissures and osteochondral interface permeability increase, bone-derived EVs may enter deep cartilage and directly alter chondrocyte metabolism. One study showed that mechanically induced EVs from subchondral bone osteocytes carried miR-23b-3p. This miRNA could target OTUD4 in chondrocytes, disrupt mitophagy, promote catabolism, and inhibit anabolism. Inhibition of miR-23b-3p in osteocytes or chondrocytes alleviated cartilage degeneration in mice (40). This study linked mechanical loading, osteocyte EVs, mitochondrial quality control, and abnormal cartilage metabolism. It provided a mechanistic explanation for some clinical phenomena where “bone pathology precedes cartilage pathology.” It also suggests that simply delivering anabolic factors to cartilage may be insufficient to halt disease. If EVs from subchondral bone continue to deliver damage instructions to cartilage, the repair effect is likely to be counteracted.

Vascular endothelial cell-derived EVs may also participate in disease progression. Healthy articular cartilage is avascular. Blood vessels in subchondral bone are strictly limited at the calcified cartilage interface. During osteoarthritis, angiogenesis and nerve ingrowth can alter oxygen tension, nutrition, and pain sensitivity. Yang et al. found that vascular endothelial cell-derived exosomes could inhibit chondrocyte autophagy and p21-related antioxidant responses. They increased reactive oxygen species (ROS) and promoted apoptosis, thereby aggravating osteoarthritis (63). This result suggests that abnormal vascularization not only damages cartilage through physical invasion and inflammatory cell transport, but may also change chondrocyte fate at a distance through EVs.

The infrapatellar fat pad is an important metabolic and immune organ within the joint. Its adipocytes, macrophages, vascular cells, and mesenchymal stromal cells can all release EVs (64). In the obese state, adipose tissue inflammation, lipotoxicity, and insulin resistance may alter the lipid and miRNA composition of EVs. This shifts them from tissue-supportive signals toward pro-inflammatory and metabolic damage signals. The infrapatellar fat pad is adjacent to the synovium and cartilage. EVs released from it can enter the synovial fluid without passing through the systemic circulation. Therefore, it may be an important mediator connecting systemic metabolism and local joint pathology in obesity-related osteoarthritis (64). On the other hand, infrapatellar fat pad mesenchymal stromal cell EVs may also retain joint tissue-adaptive protective cargo (65). This difference in cell sources within the same tissue indicates that “adipose-derived EVs” cannot be simply viewed as a single uniform category.

### 3.4 The pathological EVs cycle connects inflammation, mitochondrial damage, matrix degradation, and aberrant repair

The above evidence collectively points to a self-sustaining pathological EV cycle. Mechanical injury, aging, or metabolic abnormalities first put a small number of chondrocytes, osteocytes, and synovial cells into a stressed state. EVs released by these cells activate macrophages and fibroblasts. This causes them to undergo inflammation and metabolic reprogramming. Inflammatory synovial cells then produce more EVs, cytokines, and proteases. This further damages chondrocyte autophagy, mitochondria, and matrix synthesis. Damaged and senescent chondrocytes in turn release abnormal EVs and matrix fragments. These continuously stimulate the synovium and subchondral bone. Pathological information thus no longer depends on the persistence of the initial injury. It is mutually maintained by multiple tissue nodes (66, 67).

**Figure 1:**
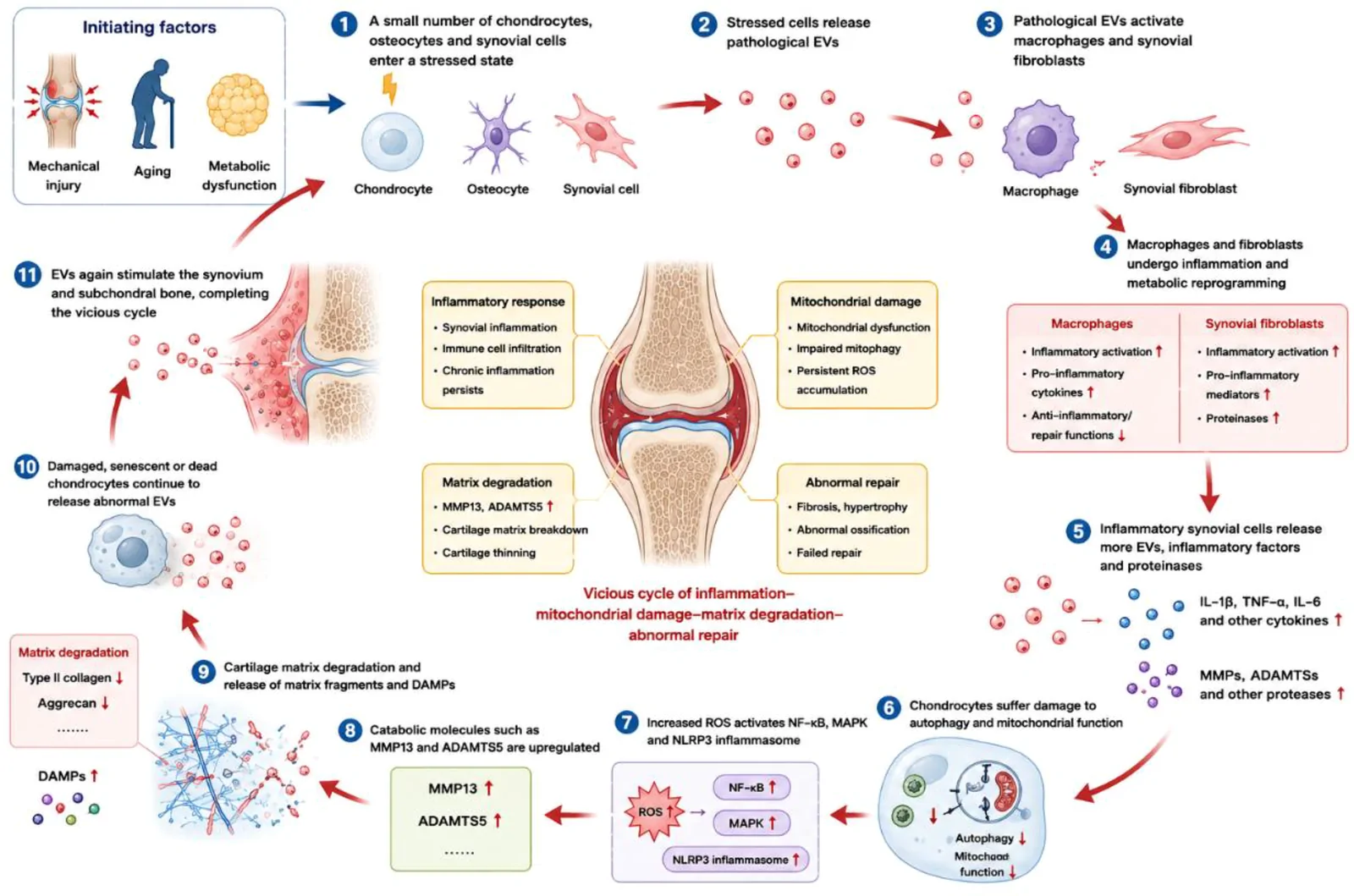
Pathological EVs drive the inflammation-mitochondrial damage-matrix degradation cycle in osteoarthritis. Pathological EVs drive the inflammation-mitochondrial damage-matrix degradation cycle in osteoarthritis. Initiating factors such as mechanical injury, aging, and metabolic dysfunction put a small number of chondrocytes, osteocytes, and synovial cells into a stressed state. They release pathological EVs with pro-inflammatory and tissue-damaging effects. These EVs can activate synovial macrophages and synovial fibroblasts. This induces inflammatory responses and metabolic reprogramming, further increasing the release of pathological EVs, inflammatory cytokines, and proteases. Sustained inflammatory signals impair chondrocyte autophagy and mitochondrial quality control. This leads to accumulation of reactive ROS and activation of NF-κB, MAPK, and NLRP3 inflammasome pathways. These pathways promote the expression of catabolic molecules such as MMP13 and ADAMTS5. The resulting degradation of chondrocyte extracellular matrix releases more matrix fragments and DAMPs. Meanwhile, damaged, senescent, or dead chondrocytes continue to release abnormal EVs. These re-stimulate the synovium and subchondral bone. This forms a cross-tissue, self-sustaining positive feedback loop. This cycle ultimately manifests as persistent synovitis and immune cell infiltration, mitochondrial dysfunction and ROS accumulation, cartilage matrix loss and cartilage thinning, and failed repair including fibrosis, chondrocyte hypertrophy, and abnormal ossification.

At the mitochondrial level, pathological EVs can inhibit mitophagy, increase ROS, promote loss of membrane potential, and shift energy metabolism. ROS can in turn activate nuclear factor-κB (NF-κB), mitogen-activated protein kinase (MAPK), and NOD-like receptor family pyrin domain containing 3 (NLRP3) inflammasomes. This increases the expression of MMP13, ADAMTS5, and inflammatory mediators (68). Collagen and proteoglycan fragments generated by matrix degradation act as DAMPs. They further activate TLRs and innate immune pathways. Meanwhile, cellular senescence, ferroptosis, apoptosis, or pyroptosis release more danger signals. This forms a mutually reinforcing loop of “EVs – mitochondrial damage – inflammation – matrix degradation” (69). This model can explain why therapies targeting a single cytokine often fail to achieve stable disease-modifying effects in heterogeneous osteoarthritis. Even if IL-1β or TNF-α is briefly blocked, pathological EVs may simultaneously activate multiple alternative pathways through miRNAs, proteins, lipids, and metabolites. Similarly, inhibiting only one MMP cannot resolve upstream synovial immune and subchondral bone signals (70). A more rational therapeutic strategy should intervene at multiple levels. This includes reducing the production of pathological EVs or abnormal cargo sorting, blocking their binding to key recipient cells, clearing pathogenic EVs, or using therapeutic EVs to override and rewrite the cell states they transmit. The pathological EV cycle may also change with disease stage. In early disease, synovial and immune cell EVs may play a more dominant role. The therapeutic window is mainly to suppress inflammatory amplification and restore chondrocyte homeostasis. In mid-stage disease, senescent cells and subchondral bone remodeling gradually become more important. It is necessary to simultaneously address cellular senescence, mitochondrial dysfunction, and the osteochondral interface. After extensive cartilage loss in late disease, natural EVs may be unable to achieve structural reconstruction relying solely on endogenous cell repair. They need to be combined with scaffolds, cells, or surgical repair (71). Therefore, “blocking the pathological EV cycle” should not be designed as a fixed single-target regimen. Instead, the main pathological nodes should be selected according to joint tissue status (**Figure 1**).

## 4 From suppressing inflammation to resetting immunity: therapeutic EVs remodel the osteoarthritis immune microenvironment

### 4.1 Modulating macrophage states and interrupting the synovium-cartilage inflammatory feedback

Early studies on mesenchymal stromal cell-derived EVs mostly focused on chondrocyte proliferation and matrix expression. However, increasing evidence suggests that macrophages may be one of the first and most critical recipients of therapeutic EVs within the joint. Macrophages rapidly phagocytose particles in the joint cavity. Changes in their state can in turn affect fibroblasts, chondrocytes, and endogenous progenitor cells through cytokines and EVs. If therapeutic EVs first reduce the pro-inflammatory and tissue-destructive programs of macrophages, they may improve the overall joint environment before the EVs actually reach deep cartilage (72). Cosenza et al. compared bone marrow mesenchymal stromal cell-derived exosomes and microEVs. They found that both could restore type II collagen and aggrecan expression in osteoarthritic chondrocytes. They also inhibited MMP13, ADAMTS5, and inducible nitric oxide synthase (iNOS) and reduced chondrocyte apoptosis and macrophage activation. In a collagenase-induced mouse osteoarthritis model, both types of EVs protected joint structure (73). This study suggests that the main chondroprotective effects of mesenchymal stromal cells can be partially replicated by different EV subpopulations. Moreover, anti-inflammatory and chondroprotective effects are not two independent effects. Zhang et al. further studied mesenchymal stromal cell exosome-mediated osteochondral repair. They found increased cell proliferation and infiltration in the repair tissue, enhanced matrix synthesis, and formation of an immune phenotype more favorable for tissue regeneration (22). This result supports the concept of “regenerative immunity.” Effective repair does not completely eliminate immune responses. Rather, it shifts immune cells from continuously producing damage signals toward clearing debris, limiting inflammation, and supporting matrix reconstruction. In other words, the goal of therapeutic EVs is not to make the joint an immunologically silent environment. Instead, it is to re-establish immune processes that initiate and resolve in a timely manner.

**Figure 2:**
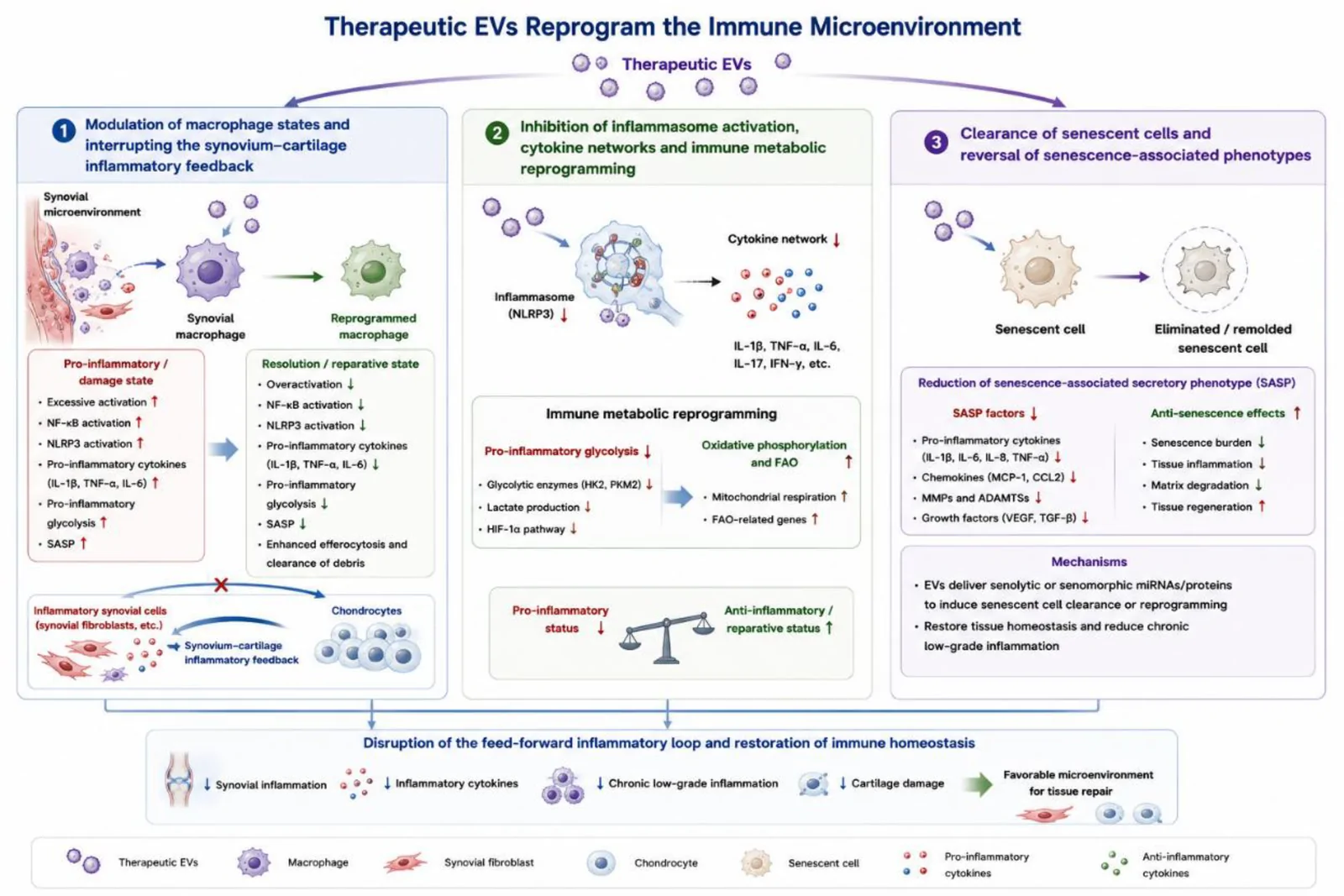
Therapeutic EVs remodel the osteoarthritis immune microenvironment. Therapeutic EVs remodel the osteoarthritis immune microenvironment. Therapeutic EVs act through three interconnected levels to resolve persistent low-grade inflammation in the osteoarthritic joint. (1) Modulating macrophage state and interrupting synovium-cartilage inflammatory feedback: EVs act on synovial macrophages. They suppress excessive immune activation, NF-κB and NLRP3 inflammasome signaling, pro-inflammatory cytokine release, pro-inflammatory glycolysis, and SASP. They also enhance efferocytosis and clearance of tissue debris. This shifts macrophages from a sustained pro-inflammatory/tissue-damaging state toward an inflammation-resolving and repair-supporting state. This thereby weakens the positive inflammatory feedback between synovial fibroblasts and chondrocytes. (2) Inhibiting inflammasomes and cytokine networks and correcting immunometabolic reprogramming: Therapeutic EVs reduce pro-inflammatory signals such as IL-1β, TNF-α, IL-6, IL-17, and IFN-γ. They inhibit pro-inflammatory glycolysis, lactate production, and HIF-1α-related metabolic programs. They also promote oxidative phosphorylation, fatty acid oxidation (FAO), and mitochondrial respiration. This shifts immune cells from a pro-inflammatory metabolic state toward an anti-inflammatory/repair metabolic state. (3) Clearing or remodeling senescent cells: EVs can deliver miRNAs and proteins with senolytic or senescence-modulating effects. They reduce SASP, chemokines, matrix-degrading enzymes, and other paracrine damage signals. They reduce senescence burden, tissue inflammation, and matrix degradation. They also relieve inhibition of local tissue regeneration. These actions together interrupt the feed-forward inflammatory loop, restore joint immune homeostasis, and establish a microenvironment favorable for subsequent tissue repair.

Macrophage states are often summarized as M1 versus M2 polarization. However, this binary model cannot cover the multiple subpopulations simultaneously exhibiting inflammatory, phagocytic, lipid metabolic, tissue-resident, interferon response, and senescent characteristics in patient synovium (28). Therefore, when observing decreased iNOS and increased CD206 in EVs studies, one should not directly infer that all macrophages have undergone stable “M2 polarization.” More reliable evaluation should combine transcriptomics, metabolic flux, cytokine profiles, phagocytosis, and efferocytosis functions. It should also confirm whether the state change is sustained and whether it truly improves cartilage outcomes (74). Nevertheless, EVs from regulatory macrophages themselves also have therapeutic potential. A study on M2-like macrophage small EVs showed that such EVs could improve chondrocyte survival, reduce apoptosis and matrix degradation, and lower IL-6 and TNF-α levels. Their effects were related to osteopontin pathway regulation (75). This result indicates that immune cell EVs are not inherently pathogenic. The key remains the donor cell state and cargo composition. Using favorable macrophage EVs as therapeutic agents, or using mesenchymal stromal cell EVs to reprogram patient synovial macrophages into a favorable state, may both interrupt the synovium-cartilage inflammatory feedback (**Figure 2**).

### 4.2 Inhibiting inflammasomes, cytokine networks, and immunometabolic reprogramming

Inflammation in the osteoarthritic synovium is usually milder than in rheumatoid arthritis. However, it is persistent, low-grade, and difficult to resolve spontaneously. TLRs, NF-κB, MAPK, and NLRP3 inflammasomes can be co-activated by matrix fragments, crystals, mitochondrial DNA, ROS, and abnormal lipid metabolism. Therapeutic EVs contain miRNAs, antioxidant enzymes, and regulatory proteins that can act on multiple nodes simultaneously (76). Therefore, they are more likely than single receptor antagonists to weaken the redundancy of the inflammatory network. For example, a 2026 study on bone marrow mesenchymal stromal cell exosomal miR-27a-3p examined the TXNIP/NLRP3 axis in both chondrocytes and macrophages. It showed that EVs enriched with miR-27a-3p could simultaneously alleviate inflammatory damage in chondrocytes and inflammatory responses in macrophages (77). This “dual recipient cell” design is closer to the joint ecosystem than studies measuring only MMP13 in chondrocytes. Its significance lies in the fact that the same EV cargo may simultaneously alter the inflammatory output of immune cells and the damage response of chondrocytes by inhibiting common oxidative stress-inflammasome nodes.

Immunometabolism is another important layer. Pro-inflammatory macrophages often enhance glycolysis and lipid synthesis to support rapid cytokine production. Inflammation resolution and tissue repair depend more on mitochondrial oxidation, fatty acid utilization, and effective efferocytosis (78). As mentioned earlier, inflammatory synovial fibroblast EVs could enhance macrophage glycolysis. Glycolysis inhibition attenuated their pro-inflammatory effects. This indicates that pathological EVs transmit metabolic state as part of the inflammatory memory. If therapeutic EVs can restore mitochondrial function, reduce TXNIP and ROS, and improve lipid metabolism, they may reduce the sensitivity of macrophages to inflammatory stimuli at the metabolic base. This is more than just transiently reducing cytokine release. Umbilical cord mesenchymal stromal cell-derived exosomes have advantages of easy expansion and low immunogenicity. Related studies have shown that human umbilical cord mesenchymal stromal cell exosomes not only promote chondrocyte proliferation and migration and inhibit apoptosis, but also regulate macrophage state and reduce articular cartilage damage in rats (79). Bone morphogenetic protein 7 (BMP-7)-modified synovial mesenchymal stromal cell-derived exosomes were used to enhance macrophage regulation and chondroprotection. Researchers observed their regulatory effects on inflammatory cytokines, iNOS, and CD206-related phenotypes (80). These results show that therapeutic EVs can alter immunomodulatory profiles through genetic modification of donor cells. However, they also suggest that the mechanism of action and safety of the final product will be more complex than those of natural EVs (**Figure 2**).

### 4.3 Clearing or remodeling senescent cells to relieve the regeneration-inhibitory microenvironment

In chronic osteoarthritis, simply suppressing inflammation may be insufficient to restore repair. This is because a large number of senescent chondrocytes, fibroblasts, and immune cells continuously produce SASP and form an inhibitory environment for regenerative cells (56). Senescent cells need not be numerous in number. They can amplify their effects through paracrine signals. Ideal EV therapy should distinguish between recoverable stressed cells and irreversibly senescent cells. The former require mitochondrial repair, autophagy restoration, and phenotype remodeling. The latter may require selective clearance (81, 82). Studies on hypoxic adipose-derived mesenchymal stromal cell exosomes have shown that therapeutic EVs can reduce chondrocyte SASP and attenuate osteoarthritis *in vivo*. FTO-overexpressing EVs can enhance autophagy, reduce ROS, and inhibit chondrocyte senescence (83, 84). Such strategies are closer to “senescence phenotype reversal” or “senescence modulation.” Their advantage is preserving chondrocytes that still have repair potential. The risk is that some cells may only transiently reduce p16, p21, or inflammatory molecules, while DNA damage and genomic instability persist (81). Therefore, long-term tracking of cell cycle, DNA damage, mitochondrial function, and tumor-related risks is needed. One should not conclude that rejuvenation has been achieved based solely on a decrease in senescence-associated β-galactosidase.

Another strategy is to engineer EVs as senescent cell clearance vehicles. A study on engineered mesenchymal stromal cell small EVs loaded siMDM2 into EVs. WYRGRL-related cartilage-targeting moieties were introduced on the surface to enhance effects on senescent chondrocytes and maintain cartilage metabolic homeostasis (85). This design embodies the logic of “targeting senescent cells – selective clearance – relieving SASP – allowing neighboring cell regeneration.” Compared with free senescent cell‑clearing agents, EVs may reduce systemic exposure. However, their effects on normal proliferating cells, chondroprogenitors, and other p53-related cells still need systematic evaluation. More notably, a 2026 randomized clinical study by Meng et al. on temporomandibular joint osteoarthritis used autologous circulating EVs. They found that compared with hyaluronic acid controls, EV treatment improved symptoms and condylar bone regeneration. No significant adverse events within the reported scope were observed. Mechanistic studies suggested that C1QBP-rich circulating EVs could selectively clear senescent chondrocytes through the C1QBP/C1q/p14ARF-related mitochondrial apoptosis pathway. They also modulated the immune environment and tissue regeneration (86). This study is an important advance in the clinical translation of EVs for osteoarthritis. However, its subjects were temporomandibular joint osteoarthritis. The joint anatomy, cartilage type, loading mode, and preparation source differ from knee osteoarthritis. The results cannot be directly extrapolated as routine therapeutic evidence for knee joints (**Figure 2**).

### 4.4 Cell source and preconditioning methods determine the immunomodulatory profile of therapeutic EVs

Bone marrow mesenchymal stromal cells, adipose mesenchymal stromal cells, synovial mesenchymal stromal cells, infrapatellar fat pad mesenchymal stromal cells, umbilical cord mesenchymal stromal cells, and dental pulp stem cells have all been used to prepare therapeutic EVs for osteoarthritis (87). Bone marrow mesenchymal stromal cells have a substantial research foundation. Their EVs can inhibit inflammation, apoptosis, and matrix breakdown. However, donor age and bone marrow status may affect yield. Adipose mesenchymal stromal cells are readily accessible and expand quickly. However, they are susceptible to obesity, insulin resistance, and harvest site. Synovial mesenchymal stromal cells have strong chondrogenic potential and joint tissue adaptability. However, patient synovitis may alter their secretion profile. Umbilical cord mesenchymal stromal cells are young, expand well, and are suitable for allogeneic large-scale production. However, it needs to be confirmed whether their efficacy can stably cover different osteoarthritis phenotypes (88). Source comparisons cannot be based only on particle yield. Multidimensional efficacy including immunosuppression, chondroprotection, matrix synthesis, vascular regulation, and bone remodeling should be compared under the same culture conditions, purification procedures, and dose units (89). Many existing studies have been conducted in different laboratories using different animal models and doses. Therefore, conclusions that “one source is superior to another” remain unstable. Comparative studies of adipose and bone marrow mesenchymal stromal cell exosomes and comparisons between exosomes and plasma membrane-derived EVs suggest that different sources and EV subtypes may differ in cartilage regeneration capacity. However, more rigorous head-to-head validation is needed (36, 90).

Donor cell preconditioning can further alter EVs. Hypoxia can mimic the environment of mesenchymal stromal cells in injured tissues. It can enhance certain pro-angiogenic, anti-apoptotic, and autophagy-related cargo. Mechanical stimulation can increase EV yield and function related to cartilage adaptation. Inflammatory cytokine preconditioning may enhance immunomodulatory cargo. However, there is also a risk of introducing pro-inflammatory components. A study on mechanical stimulation of umbilical cord mesenchymal stromal cells found that mechanical loading could increase exosome yield and repair capacity. Its effects were related to enrichment of lncRNA H19 (91). This study shows that the physical environment during manufacturing can be considered a bioreactor parameter, not merely an irrelevant culture detail. In addition, a study on mechanical load-preconditioned mesenchymal stromal cells found that cyclic stretching enriched miR-330-3p in EVs. This cargo could improve chondrocyte mitochondrial quality control through FKBP4-FoxO3a and PINK1/Parkin-related mitophagy. It promoted proliferation and migration and inhibited apoptosis, senescence, and matrix degradation (92). This study linked mechanical preconditioning, EV cargo changes, and organelle repair. It showed that preconditioning can produce “adaptive EVs” with defined mechanisms. However, in clinical manufacturing, mechanical amplitude, frequency, timing, and cell density must all be standardized. Otherwise, batch-to-batch cargo differences may further widen (**Table 1**).

**Table 1:** Comparison of effects and translational characteristics of EVs from different cell sources in osteoarthritis.

EV source	Core effects and mechanisms	Main advantages	Key limitations	Suitable applications	References
Bone marrow MSC	Restore COL2A1/ACAN; inhibit MMP13/ADAMTS5/iNOS; anti-apoptosis; inhibit ferroptosis	Solid research foundation; strong anti-inflammatory and chondroprotective evidence	Invasive bone marrow harvest; low yield; significant donor age and passage variation	Basic anti-inflammatory, anti-cell death agents	(73, 93)
Adipose MSC	Inhibit ER stress, apoptosis, and senescence; regulate autophagy and matrix metabolism	Abundant tissue source; high yield; easy for large-scale production and engineering	Obesity and metabolic disease alter EV cargo; weak natural cartilage targeting	Large-scale production; combination with hydrogels and nucleic acid delivery	(36, 94, 95)
Synovial MSC	Promote chondrocyte migration/proliferation; miR-140-5p restores SOX9 and matrix synthesis via RalA	Joint tissue-adapted; strong chondrogenic potential	Harvest requires arthroscopy; inflammatory state may introduce unfavorable cargo	Early-to-mid cartilage injury and progenitor cell activation	(96)
Infrapatellar fat pad MSC	Potent inhibition of matrix degradation; advantages related to miR-99 family regulating ADAMTS4	Joint-intrinsic; strong microenvironmental adaptability	Limited tissue availability; cell function in late OA donors may shift	Mechanism reference and key protective cargo screening	(37, 97)
Umbilical cord MSC	Promote chondrocyte proliferation/migration; inhibit apoptosis; regulate macrophage state	Young donors; robust expansion; suitable for allogeneic standardized production	Allo-immunogenicity assessment needed; lack of natural cartilage homing	Clinical-grade allogeneic off-the-shelf formulations and surface engineering platforms	(79, 98, 99)
Induced pluripotent stem cell MSC	Promote chondrocyte migration, proliferation, and type II collagen expression; superior to synovial MSC-EV	Unlimited expansion; high batch-to-batch consistency; reduced donor variability	Long manufacturing chain; strict exclusion of tumorigenic risk	Highly standardized “off-the-shelf” products, but with stricter safety controls	(100)
Chondrocyte-derived EV	miR-95-5p targets HDAC2/8, promoting chondrogenesis and matrix expression	Highly homologous to chondrocytes; retains cartilage-specific signals	Limited cell expansion; prone to dedifferentiation *in vitro*; pathological EVs have pathogenic effects	Discovering cartilage-specific protective targets; not suitable as general-purpose product	(54)
Repair-type macrophage-derived EV	Reduce chondrocyte apoptosis and matrix degradation; osteopontin pathway involved	Directly targets synovial immune imbalance and macrophage feedback loop	M1/M2 binary model cannot represent complex *in vivo* subpopulations; state maintenance unstable	Stage-specific immune reset in inflammatory OA	(75, 101)
Pathological source EV (pro-inflammatory fibroblast/macrophage/osteocyte)	Pro-inflammatory fibroblast EVs enhance macrophage glycolysis; pro-inflammatory macrophage EVs deliver miR-155-5p inhibiting autophagy; osteocyte EVs deliver miR-23b-3p disrupting mitophagy	Identifying pathological targets, disease markers, and blocking strategy development	Pathogenic per se; not suitable as therapeutic agents	Interventions to clear, block, or target pathological cells	(21, 34, 40)

## 5 From cytoprotection to regenerative instructions: EVs drive cartilage and osteochondral unit repair

### 5.1 Restoring the anabolic-catabolic balance of chondrocytes

**Figure 3:**
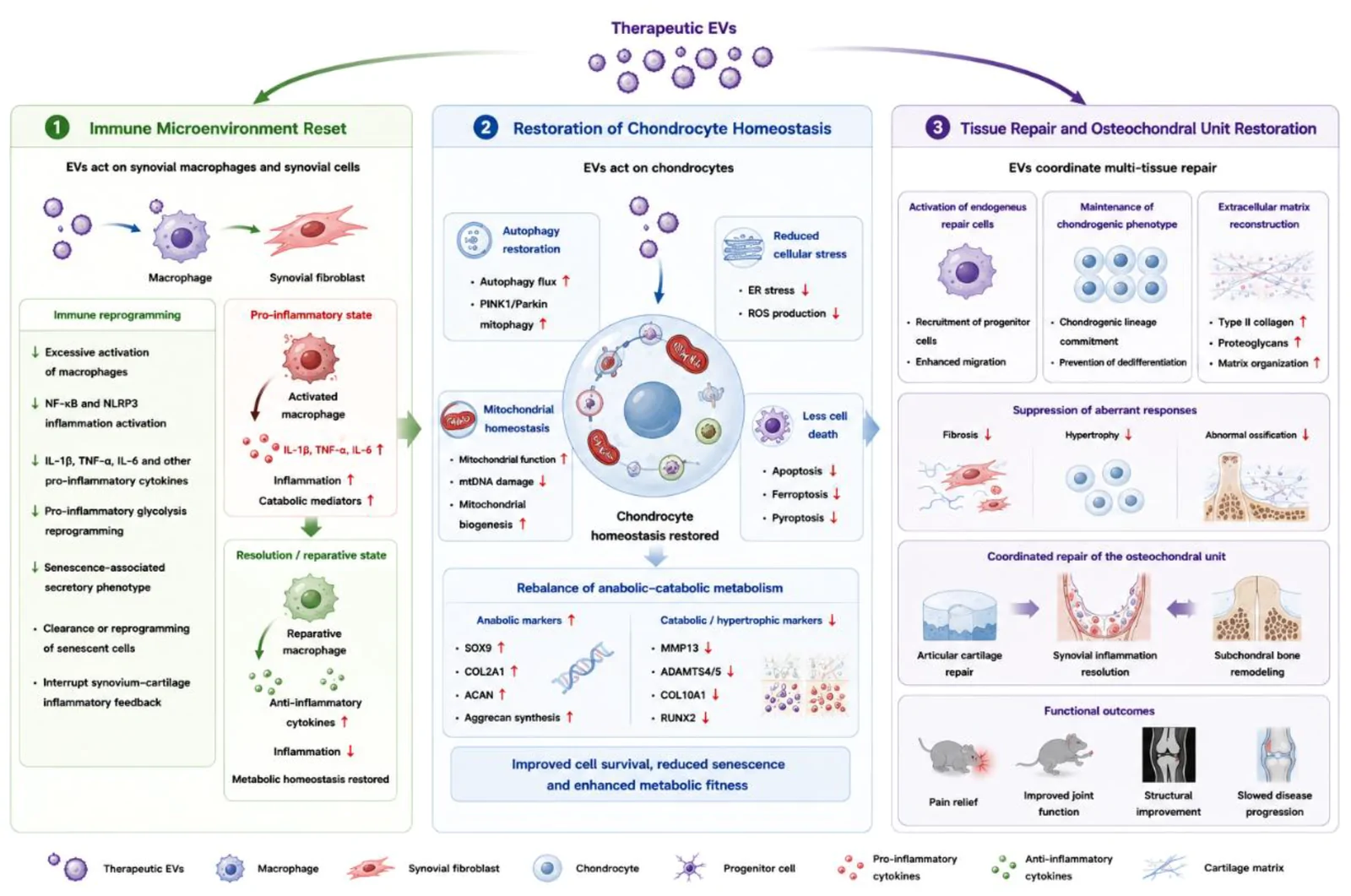
Evs drive cartilage and osteochondral unit repair. EVs drive cartilage and osteochondral unit repair. Therapeutic EVs promote the transition of osteoarthritis from a persistent injury state to a regenerative state through three interconnected levels: immunity, cell homeostasis, and tissue repair. (1) Immune microenvironment resetting: EVs act on synovial macrophages and synovial cells. They reduce macrophage overactivation, NF-κB/NLRP3 inflammatory signaling, pro-inflammatory cytokines such as IL-1β, TNF-α, and IL-6, pro-inflammatory glycolysis, and SASP. They also promote inflammation resolution, senescent cell clearance or remodeling, and metabolic homeostasis recovery. This interrupts the synovium-cartilage inflammatory feedback. (2) Restoring chondrocyte homeostasis: EVs enhance autophagy and PINK1/Parkin-related mitochondrial quality control. They improve mitochondrial function and reduce ER stress and ROS production. They also inhibit programmed cell death including apoptosis, ferroptosis, and pyroptosis. At the metabolic level, they upregulate cartilage anabolic programs such as SOX9, COL2A1, ACAN, and aggrecan synthesis. They downregulate catabolic and hypertrophic programs including MMP13, ADAMTS5, COL10A1, and RUNX2. This improves chondrocyte survival, reduces senescence, and restores the anabolic-catabolic balance. (3) Tissue repair and osteochondral unit restoration: EVs further promote endogenous progenitor cell recruitment and migration, maintain chondrogenic phenotype, and reconstruct extracellular matrix centered on type II collagen and proteoglycans. They also inhibit fibrosis, abnormal hypertrophy, and abnormal ossification. They coordinate articular cartilage repair, synovial inflammation resolution, and subchondral bone remodeling. These effects ultimately manifest as pain relief, improved joint function, structural repair, and slowed disease progression.

The minimum requirement for cartilage regeneration is not short-term survival of chondrocytes. It is restoration of the chondrocyte phenotype maintained by SOX9, COL2A1, ACAN, and others. It also requires inhibition of catabolic or hypertrophic programs such as MMP13, ADAMTS4/5, COL10A1, and RUNX2 (38, 102, 103). Many EV studies only observe cell proliferation and decreased inflammatory cytokines. If they do not demonstrate that the newly formed matrix has hyaline cartilage characteristics, correct collagen fiber arrangement, and long-term mechanical properties, then it cannot be equated with functional cartilage regeneration. A classic study used miR-140-5p-overexpressing synovial mesenchymal stromal cell-derived exosomes to observe improvement in cartilage regeneration and osteoarthritis progression (96). This suggests that cartilage-enriched miRNAs can be loaded into tissue-associated EVs to enhance efficacy. Non-coding RNAs can also influence chondrocytes through ceRNA networks. One study found that KLF3-AS1 could promote chondrocyte proliferation and alleviate osteoarthritis-like changes through the miR-206/GIT1-related axis (104). Subsequent studies have continued to show that exosomal lncRNAs may function by adsorbing miRNAs and regulating multiple gene networks (105). However, the ceRNA mechanism requires sufficient molecular abundance and subcellular co-localization. Future studies should quantitatively determine lncRNA copy numbers obtained from single EVs or single cells. This will avoid overestimating its *in vivo* contribution based only on expression correlation and dual-luciferase assays. Besides RNA, EV proteins and lipids may also directly regulate recipients. SOD3-enriched exosomes were loaded into hydrogel microspheres to enhance antioxidant and chondroprotective capacity. Related studies combined single-cell data with material delivery. This reflects the approach of screening functional proteins from disease pathways, then enhancing local efficacy through EVs and materials (106). Future regeneration research needs to shift from “which miRNA is most highly expressed” to multi-component efficacy maps. It should clarify whether RNA, protein, and lipids are synergistic, redundant, or antagonistic (**Figure 3**).

### 5.2 Regulating autophagy, mitochondrial homeostasis, and multiple programmed cell death pathways

Chondrocytes reside in a long-term hypoxic and high-load environment. Autophagy is an important mechanism for clearing damaged proteins and organelles and maintaining energy balance. With aging and osteoarthritis progression, autophagic capacity declines. Damaged mitochondria accumulate. ROS and mitochondrial DNA leakage increase. This then activates inflammation and cell death (107, 108). The protective effects of therapeutic EVs on cartilage are increasingly attributed to restoration of autophagy and mitochondrial quality control. This is not merely inhibition of extracellular inflammatory cytokines (109–111). A study on engineered subcutaneous adipose mesenchymal stromal cell exosomes found that EV-enriched miR-199a-3p could regulate the mTOR-autophagy pathway and alleviate osteoarthritis in rats and mice. When a chondrocyte-affinity peptide was linked to the EV surface, it enhanced the ability to enter cartilage tissue and deliver miR-199a-3p (112). This study embodies the combination of natural cargo loading mechanisms and targeting engineering. First, an endogenous miRNA beneficial to cartilage was identified in EVs. Then the problem of insufficient delivery to recipient cells *in vivo* was addressed. miR-122-5p in hypoxic mesenchymal stromal cell EVs was also shown to regulate ERK1/2 and p38-related signaling and chondrocyte autophagy by targeting DUSP2. This promoted chondrocyte regeneration (113). This study validated the effects of hypoxic EVs *in vitro* and in a rat osteoarthritis model. Its implication is that the MAPK pathway is not simply a pro-inflammatory switch. It has context-dependent relationships with autophagy, proliferation, and stress adaptation. When evaluating EV mechanisms, one should examine the amplitude, duration, and subcellular location of signaling activation. One should not judge benefit or harm solely based on whether phosphorylation is increased or decreased.

Endoplasmic reticulum (ER) stress and mitochondrial stress interact and can also lead to chondrocyte apoptosis. A study on miR-486-5p-modified adipose-derived mesenchymal stromal cell exosomes showed that miR-486-5p-enriched EVs had better intra-articular persistence than free miRNA and miRNA-overexpressing cells. They also more effectively alleviated chondrocyte ER stress and osteoarthritis. *In vivo* imaging showed that their intra-articular signal could be maintained for several days. This indicates that the EV membrane not only mediates targeting but also protects nucleic acid cargo and prolongs local exposure (94).

In addition to apoptosis, ferroptosis, pyroptosis, and necroptosis are gradually being incorporated into the cartilage cell death atlas. Ferroptosis is driven by iron-dependent lipid peroxidation. It is closely related to ACSL4, GPX4, glutathione, and mitochondrial metabolism. A bone marrow mesenchymal stromal cell exosome study found that they could reduce chondrocyte ferroptosis and delay osteoarthritis by intervening in the METTL3-m6A-ACSL4 axis (93). This indicates that EVs can influence lipid metabolism and cell death by regulating RNA methylation, rather than merely providing antioxidant molecules directly. One study proposed a “reverse adaptation” strategy. Osteoarthritis-like oxidative stress was used to precondition mesenchymal stromal cells. This enriched their EVs with anti-ferroptotic miR-142a-3p. This was then combined with engineered delivery to enhance therapy (114). This study shows that moderate pathological stimulation can train donor cells to generate more targeted EVs. However, excessive preconditioning intensity may cause mesenchymal stromal cell damage, DNA abnormalities, or increased pro-inflammatory cargo. Therefore, it is necessary to establish a process design space linking stimulation range, cell state, and final EV efficacy (**Figure 3**).

### 5.3 Coordinating coupled repair of cartilage, subchondral bone, blood vessels, and synovium

Histological improvement in cartilage does not necessarily mean recovery of the entire joint. If subchondral bone continues to exhibit high turnover or sclerosis, the synovium remains chronically inflamed, and abnormal blood vessels continue to invade, then locally regenerated cartilage may degenerate again. Osteoarthritis EV therapy needs to upgrade from “chondrocyte protection” to “combined repair of the osteochondral unit and synovium.” This requires EVs to both maintain cartilage matrix and regulate osteoblast-osteoclast balance, angiogenesis, synovial fibrosis, and mechanical adaptation. Subchondral bone repair is biphasic. In early disease, inhibiting excessive bone resorption and abnormal vascularization may be beneficial. In late disease with trabecular bone destruction or bone defects, moderate bone formation is needed (115). If EVs broadly promote angiogenesis, this may benefit bone repair. However, it may exacerbate cartilage vascular invasion and nerve growth. Therefore, the same “pro-angiogenic” cargo cannot be universally regarded as a regenerative advantage. Its effects depend on the delivery site and disease stage. An ideal system should maintain a low-vascular environment in the cartilage layer while promoting controlled vascular-osteogenic coupling in the subchondral bone layer. Synovial repair is also important. Persistent synovial fibrosis alters joint compliance, synovial fluid composition, and EV transport. It may also become a source of pain. If therapeutic EVs only reduce inflammation but do not prevent the transformation of fibroblasts into myofibroblasts, long-term structural benefits may be limited. Future studies should simultaneously detect synovial thickness, fibrosis markers, macrophage subpopulations, cartilage structure, subchondral bone parameters, and pain behavior. They should analyze the temporal relationships among these outcomes. This will determine which tissue node the EVs primarily act on (**Figure 3**).

## 6 From natural EVs to definable biologics: engineered delivery and clinical translation

### 6.1 Enhancing EVs function through donor cell preconditioning and cargo editing

**Figure 4:**
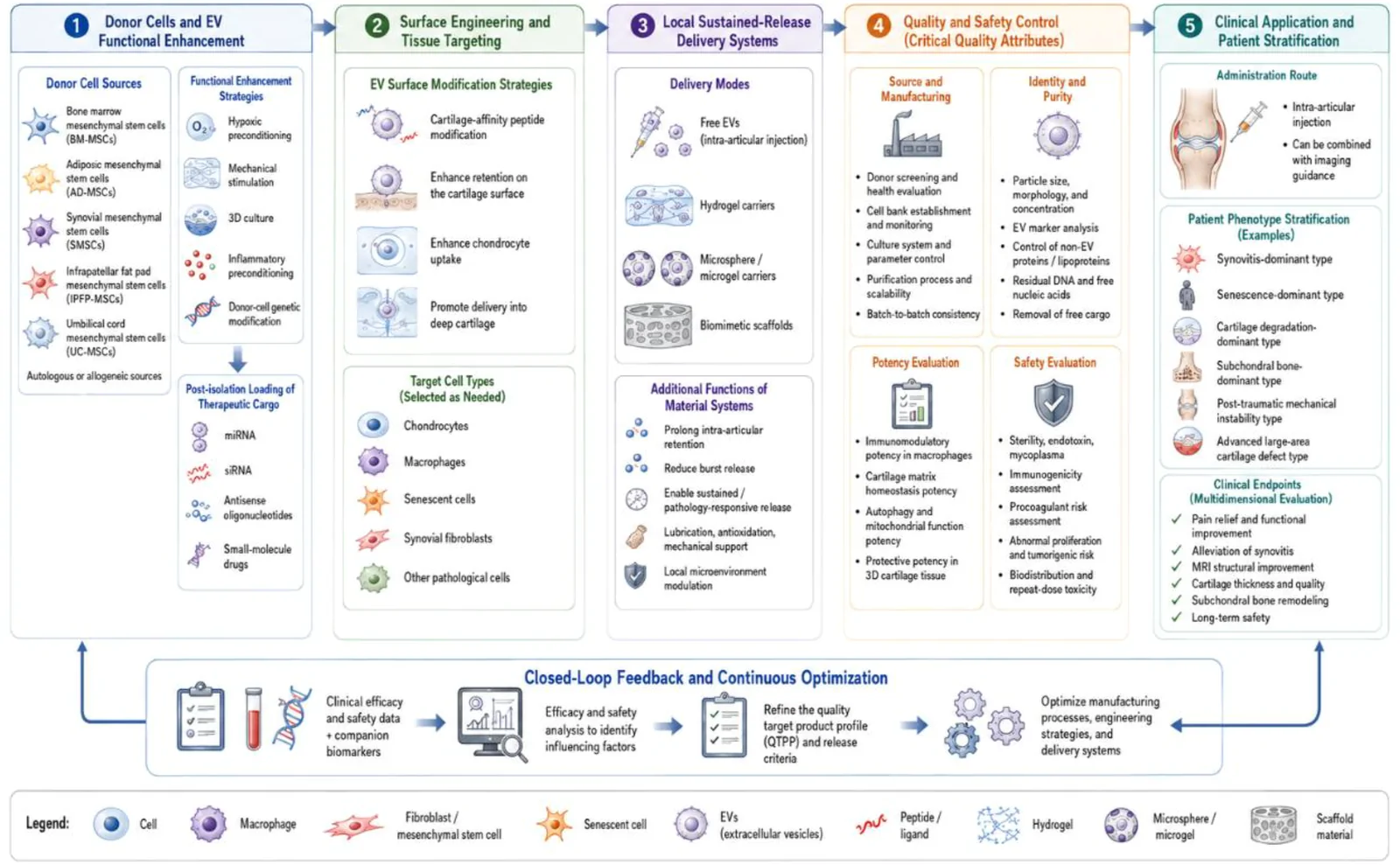
Engineering, delivery, and clinical translation workflow for osteoarthritis EV preparations. Engineering, delivery, and clinical translation workflow for osteoarthritis EV preparations. The development of OA EV preparations can be summarized into five interconnected and iterative steps. (1) Donor cell selection and EV functional enhancement: Select mesenchymal stromal cells from bone marrow, adipose, synovium, infrapatellar fat pad, or umbilical cord according to product positioning. Use hypoxia preconditioning, mechanical stimulation, three-dimensional culture, inflammatory preconditioning, or donor cell genetic modification to adjust EV yield and functional cargo. After isolation, load miRNAs, siRNAs, antisense oligonucleotides, or small molecule drugs. (2) Surface engineering and tissue/cell targeting: Enhance EV retention on cartilage surface, deep tissue penetration, and chondrocyte uptake through cartilage-affinity ligands, surface charge, or other membrane modifications. Selectively target chondrocytes, macrophages, senescent cells, synovial fibroblasts, or other pathogenic cells according to the main pathological phenotype. (3) Local sustained delivery: EVs can be injected intra-articularly directly, or combined with hydrogels, microspheres/microgels, or biomimetic scaffolds to prolong intra-articular retention, reduce burst release, achieve sustained or pathology-responsive release, and add lubrication, antioxidant, mechanical support, and local microenvironment regulation functions. (4) Quality and safety control: Establish critical quality attributes around source and production process, particle identity and purity, functional cargo, potency, and safety. These include donor and cell bank control, particle size/morphology/concentration and EV markers, free cargo and residual impurities, potency assays such as macrophage immunomodulation and cartilage homeostasis, as well as sterility, endotoxin, mycoplasma, immunogenicity, procoagulant risk, abnormal proliferation, systemic biodistribution, and repeat-dose toxicity evaluation. (5) Clinical application and patient stratification: Intra-articular injection is the main route of administration. Select more appropriate products and delivery strategies according to phenotypes such as synovitis-predominant, senescence-predominant, cartilage degradation-predominant, subchondral bone abnormality, post-traumatic mechanical instability, or large cartilage defects. Efficacy evaluation should simultaneously cover pain and function, synovitis, cartilage structure and quality, subchondral bone remodeling, and long-term safety. Clinical efficacy, safety, and concomitant biomarker data feedback to target product characteristics, release specifications, production process, engineering schemes, and delivery systems. This forms a closed translational pathway of “product design – manufacturing quality control – clinical validation – feedback optimization”.

Natural EVs have the advantages of biocompatibility and multi-component synergy. However, their effective cargo content is often low, and batch-to-batch variation is large (116). Enhancement strategies can be divided into donor cell preconditioning, donor cell genetic modification, and post-isolation direct loading. Hypoxia, mechanical stimulation, three-dimensional culture, inflammatory preconditioning, and drug treatment can adjust cargo while preserving natural sorting mechanisms. Transfection of miRNA, lncRNA, or protein expression vectors can enrich specific molecules. Electroporation, sonication, extrusion, freeze-thaw, and chemical transfection can load nucleic acids or small molecules after EV isolation (117). Donor cell engineering generally utilizes the cell’s own sorting and membrane assembly processes. This reduces physical disruption to the EV membrane. However, transfection efficiency, the proportion of cargo entering EVs, and residual plasmid or viral vector need to be controlled. Post-isolation loading facilitates dose adjustment. However, it may cause EV aggregation, membrane damage, or residual free drug (118). For example, loading miR-486-5p, miR-199a-3p, siMMP13, ASO-MMP13, or siMDM2 into EVs has shown therapeutic potential. However, the “cargo load per particle” for different loading methods is often not fully reported. This makes it difficult to compare the true efficacy of the preparations (13, 103, 119). A study on surface-functionalized exosomes delivering siMMP13 used lipid insertion to attach a cartilage-affinity peptide and loaded MMP13 siRNA inside the EVs. After intra-articular administration, the engineered EVs reduced MMP13 and increased COL2A1 and proteoglycan levels (120). A Sortase A-engineered EV study was used to deliver MMP13 antisense oligonucleotides to chondrocytes (121). These studies show that EVs can serve as nucleic acid drug platforms. However, the long-term safety of MMP13 inhibition, the need for normal cartilage remodeling, and off-target distribution still need evaluation. Multi-cargo or multi-functional engineering may be more suitable for complex osteoarthritis. However, complexity will rapidly increase manufacturing and regulatory difficulty. For each additional component such as natural EVs, targeting peptides, nucleic acid drugs, hydrogels, and gene editing tools, independent control of raw materials, purity, stability, and safety is required (122). Engineering design should be centered on addressing clear clinical bottlenecks. Examples include insufficient deep cartilage delivery, rapid intra-articular clearance, or insufficient dosage of key cargo. It should not add components without limit for the sake of technical novelty (**Figure 4**).

### 6.2 Surface engineering and cartilage targeting to improve tissue penetration and receptor selectivity

Intra-articular administration does not equate to entry into intact articular cartilage. Normal articular cartilage is a tissue environment highly restrictive to particle transport. The dense collagen network creates significant steric hindrance. The glycosaminoglycans in aggrecan give cartilage a high fixed negative charge density. Natural EVs typically also have a net negative surface charge. Therefore, their migration in normal cartilage is limited by both steric hindrance and electrostatic repulsion. Studies on unmodified MSC-sEVs have found that their ability to enter cartilage is relatively limited. Adjusting the surface charge of EVs to a moderately cationic state can improve cartilage uptake, tissue penetration, and intra-articular retention (123). EV transport in cartilage actually requires a balance between matrix binding and free diffusion. If the affinity of EVs for the matrix is too weak, particles easily re-enter the synovial fluid and are rapidly cleared. If binding is too strong, EVs may mainly remain in the superficial cartilage layer and fail to continue diffusing to deeper layers. Recent studies using cationized exosomes in healthy and early OA cartilage further support this view. Moderate increases in positive charge can enhance cartilage penetration through reversible electrostatic interactions. However, excessively strong electrostatic attraction may actually limit particle diffusion to deeper layers (124).

Targeting ligand modification is another method to improve cartilage penetration. For example, chondrocyte-affinity peptide-modified exosomes carrying miR-140 could enter deeper cartilage regions compared with unmodified EVs and enhance therapeutic cargo delivery (125). However, most current cartilage penetration studies use engineered EVs, cartilage explants, or early OA tissues for validation. There is still a lack of sufficient quantitative evidence on whether unmodified natural therapeutic EVs can efficiently penetrate the full thickness of normal adult human intact articular cartilage. Disease severity further complicates this process. As OA progresses, proteoglycan loss and matrix structural disruption may increase tissue physical permeability. However, at the same time, healthy chondrocytes and intact matrix architecture are continuously lost. Therefore, observing increased EV penetration in severely degenerated cartilage should not be automatically interpreted as a larger therapeutic window for regeneration. Future studies should simultaneously quantitatively analyze EV concentration at different depths, spatial distribution of intact particles versus released cargo, uptake efficiency of chondrocytes in different cartilage zones, and true functional cargo activity (**Figure 4**).

### 6.3 Hydrogels, microspheres, and biomimetic scaffolds to prolong intra-articular retention and achieve sustained release

Joint movement, synovial fluid exchange, phagocytosis, and lymphatic drainage cause rapid clearance of free EVs. Repeated intra-articular injections increase infection risk, compliance issues, and cost. Hydrogels, microspheres, scaffolds, and biomimetic extracellular matrices can immobilize EVs locally. They reduce burst release and prolong the duration of action (126). Materials can also provide lubrication, antioxidant, mechanical support, or microenvironment-responsive functions. This transforms EVs from short-term biological signals into sustained tissue repair systems (127). An ideal carrier should have appropriate fluidity at injection, gel rapidly after entering the joint, and maintain structural stability under physiological loads. Its degradation products should be safe. Porosity and charge should allow gradual EV release without disrupting the EV membrane or inactivating cargo. The degradation rate of the material also needs to match the disease stage. Too fast a degradation cannot prolong efficacy. Too slow may hinder tissue exchange, cause foreign body reactions, or interfere with joint movement. A study on a co-delivery hydrogel of bone marrow mesenchymal stromal cell exosomes and icariin found that EVs could enhance drug uptake. The two synergistically reduced MMP13. The hydrogel prolonged intra-articular retention and improved cartilage thickness (128). A study on infrapatellar fat pad mesenchymal stromal cell exosome hydrogel combined tissue source selection, miRNA enhancement, and injectable hydrogel for sustained ADAMTS4 inhibition and matrix repair (37). These results suggest that EVs are not only therapeutic cargo but can also serve as synergistic delivery vehicles for conventional small molecule drugs. Microgels and microspheres have larger specific surface areas and more flexible injectability. They can form dispersed depots within the joint. SOD3-enriched EV microspheres, FGF18 gene-activated hybrid EV microgels, and senescent cell-clearing biomimetic microgels all reflect the direction of pathology-responsive materials development (129). Future carriers could adjust EV release based on ROS, MMPs, pH, or inflammatory enzyme levels. This would deliver higher doses to areas with greater inflammation. However, pathology-responsive systems must demonstrate specificity in the complex environment of patient joint fluid. They should avoid premature release during normal movement or mild inflammation. Materials can also serve a lubricating function. After damage to the lubricating layer on the osteoarthritic cartilage surface, shear stress increases. This further activates chondrocyte inflammation and catabolism. Combining EVs with adhesive, lubricating hydrogels could simultaneously reduce mechanical stimulation and provide biological signals. This forms a “mechanical protection – immune modulation – cell regeneration” trinity strategy. It may be more in line with the mechanical-inflammatory coupling characteristics of osteoarthritis than simply increasing EV concentration (**Figure 4**).

### 6.4 Establishing systems for source, dose, quality attributes, potency assays, and safety evaluation

Clinical translation of EVs first faces definitional issues. Different studies use ultracentrifugation, polymer precipitation, size-exclusion chromatography, tangential flow filtration, immunoaffinity, or combined methods. The purity, recovery, protein contamination, and subpopulation composition of the resulting particles differ significantly (130). If the product contains large amounts of soluble proteins, lipoproteins, or media-derived particles, the observed effects cannot all be attributed to EVs. Clinical-grade products require clear specifications for cell banks, culture systems, serum-free or EV-depleted media, collection time, purification steps, sterility and endotoxin control, and storage and transport conditions (131). Dose units are also currently inconsistent. Some use total protein amount, others use particle number, donor cell number, or conditioned medium volume. Total protein is easily affected by non-vesicular protein contamination. Particle number cannot reflect effective subpopulations and cargo. Different instruments for small particle counting show significant differences. A more reasonable approach is to simultaneously report particle number, protein amount, key cargo, and biological potency. The relationships between these indicators and *in vivo* exposure and efficacy should be established (130). Joint cavity volume, animal body weight, and cartilage surface area should also be included in cross-species dose conversion. Identity and purity testing cannot rely only on positivity for CD9, CD63, CD81, and TSG101. It should combine particle morphology, size distribution, membrane structure, EV-associated proteins, negative markers for organelle contamination, residual DNA, host cell proteins, media components, and aggregate detection.

For engineered products, targeting ligand density, loading efficiency, free cargo, membrane integrity, and long-term stability also need to be determined (132). Even if conventional particle morphology and marker expression appear to be well preserved, the storage process of EVs can still change particle recovery and biological function. Systematic studies have shown that long-term -80 °C storage and repeated freeze-thaw can reduce recoverable particle concentration, alter detected particle size, and promote particle aggregation or EV membrane fusion (133). Comparative studies of different storage conditions such as 4 °C, -20 °C, and -80 °C similarly indicate that storage temperature and time may affect EV quantity, cargo composition, cellular uptake, and functional performance (134). Therefore, there is no single universally optimal storage condition applicable to all EV products. For osteoarthritis therapeutic EV products, stability evaluation should simultaneously detect particle concentration and size, targeting ligand stability for engineered products, and most importantly, biological potency at predetermined storage time points. One cannot simply assume that the EV product remains stable just because CD9/CD63/CD81 signals are still detectable or EV-like structures are still seen under electron microscopy. The truly clinically meaningful stability criterion is whether the intended biological activity is still maintained.

Potency testing is central to determining whether EVs can become controllable drugs. A single chondrocyte proliferation assay cannot cover the immunomodulatory and regenerative mechanisms of the product. For osteoarthritis EVs, a potency matrix containing multiple indicators can be established. These include macrophage inflammation and metabolic reprogramming, chondrocyte COL2A1/ACAN versus MMP13/ADAMTS5 balance, autophagy and mitochondrial function, senescence and cell death, matrix synthesis in three-dimensional cartilage tissues, and cross-tissue effects in osteochondral co-cultures. Key indicators should be related to the proposed mechanism of action. They should be able to distinguish high-efficacy from low-efficacy batches. Studies on potency testing for biological products emphasize that potency assays should predict the therapeutic activity of the drug substance, not merely describe the product (135). A study on clinical-grade umbilical cord mesenchymal stromal cell small EVs attempted to establish reproducible, well-characterized production batches and evaluate potency and safety for osteoarthritis treatment. This is an important step from laboratory EVs to clinical-grade products (99). However, even if size, markers, and miRNA profiles are similar, it does not guarantee that different batches have the same function. Therefore, it is necessary to link process parameters, critical quality attributes, and potency results. This forms a quality-by-design manufacturing system.

Regarding safety, EVs are generally considered to have lower risks of embolism and abnormal differentiation than intact cells. However, “cell-free” does not mean risk-free. EVs may carry pro-coagulant phospholipids, tissue factor, abnormal nucleic acids, pro-fibrotic or pro-angiogenic molecules. Engineered nucleic acids may enter non-target tissues. Repeated intra-articular injections may elicit immune responses. Long-term effects of promoting proliferation and inhibiting cellular senescence also need to exclude tumor risk (136). Donor cell genetic stability, virus and mycoplasma testing, animal-derived components, immunogenicity, reproductive toxicity, and long-term biodistribution should all be included in the evaluation.

Safety evaluation of therapeutic EVs should be route-specific, source-specific, and engineering method-specific. First, local immune responses after repeated intra-articular injections should be more systematically evaluated. Although mesenchymal stromal cell-derived EVs generally have immunomodulatory effects, EV membrane proteins, donor-related antigens, abnormal lipid components, engineered surface molecules, and residual impurities from the production process may still elicit unintended immune recognition. The risk has not been adequately characterized (137). This issue may be more important for allogeneic preparations and products requiring repeated administration, especially when the osteoarthritic synovium itself is in an inflammatory state. Second, procoagulant activity should be evaluated as an important hemocompatibility quality attribute. Mesenchymal stromal cells and their derived EVs can expose phosphatidylserine and carry tissue factor (TF). Both can provide surfaces for procoagulant reactions. Their levels are affected by tissue source, cell passage number, culture conditions, and EV subpopulations (138). Although intra-articular injection significantly reduces the chance of direct contact of EVs with the blood circulation compared with intravenous infusion, the osteoarthritic synovium is vascular-rich. Some injected particles may still leave the joint through synovial vessels or the lymphatic system. Third, after surface modification, cargo engineering, or repeated administration, the risk of non-targeted systemic biodistribution should receive more attention. The *in vivo* distribution of EVs is affected by particle properties, surface ligands, administration route, tissue barriers, and clearance by the mononuclear phagocyte system. Surface modifications to enhance cartilage or specific cell uptake may also alter uptake by non-target organs (139). Pharmacokinetic and biodistribution studies should use orthogonal tracing methods that can distinguish intact EVs, free lipophilic fluorescent dyes, and released cargo. They should quantitatively evaluate intra-articular retention, the proportion entering blood vessels or the lymphatic system, and major organ exposure. For engineered EVs carrying potent miRNAs, siRNAs, gene-regulatory proteins, or senolytic cargo, functional effects in non-target tissues should also be evaluated, not just particle distribution. Fourth, EVs produced by genetically engineered donor cells introduce additional chemistry, manufacturing, and control risks. Plasmids, viral vectors, gene-editing reagents, or transgene products used to modify donor cells, if not adequately verified for clearance, could theoretically remain in the cell bank, conditioned medium, or final EV preparation. It should be emphasized that this evidence should be understood as a manufacturing and quality control risk principle. It is not evidence that corresponding adverse events have been proven for intra-articular injection of engineered EVs. Depending on the specific engineering platform, sensitive PCR-based methods should be used to detect residual plasmid/vector DNA, detect relevant vector proteins or free engineering reagents, conduct replication-competent virus testing where applicable, and continuously monitor the genetic stability of the master and working cell banks (**Figure 4**; **Table 2**).

**Table 2:** Key quality attributes and recommended evaluation systems for clinical translation of EVs preparations.

Key quality attribute	Core evaluation content	Key control and release recommendations	Significance for osteoarthritis therapy
Donor and source cell identity	Donor health status, metabolic disease, harvest site; cell phenotype confirmation	Establish stringent donor screening criteria; confirm surface markers and differentiation capacity per MSC minimum criteria	Age, obesity, and inflammatory state are core variables for batch‑to‑batch variability
Cell banking system	Cell genetic stability, passage number, senescence degree, and microbial contamination	Establish MCB/WCB; test karyotype, senescence markers, sterility, and mycoplasma	High‑passage or senescent cells may reduce EV activity and introduce aberrant cargo
Culture system	Medium, serum/alternative source, and EV‑depletion efficacy	Prioritize animal‑free culture; verify clearance efficiency of EV‑depleted serum	Heterologous EVs and lipoproteins in serum can severely interfere with purity and effect assessment
Culture and preconditioning processes	Cell density, oxygen concentration, mechanical stimulation, inflammatory preconditioning, etc.	Establish fixed process parameter ranges; establish links between preconditioning intensity and EV efficacy	Specific preconditioning can enrich beneficial cargoes, but excessive stimulation carries risk of introducing pro‑inflammatory components
Isolation and purification process	Recovery, purity, scalability, and subpopulation selectivity	For clinical scale, recommend TFF or SEC; validate effects of each step on particle integrity and potency	Products from different isolation methods are not interchangeable; methods must be strictly defined
Identity and physical attributes	Size distribution, concentration, morphology, and EV transmembrane/cytosolic markers	Use NTA/TRPS + electron microscopy; test CD9/CD63/CD81 and source cell‑related markers	Size alone is insufficient; source markers aid confirmation of batch consistency
Purity and process impurities	Free proteins, lipoproteins, non‑EV particles, aggregates, and residual DNA	Test negative markers such as ApoA1/B, albumin, GM130, calnexin; control particle/protein ratio	Lipoproteins and soluble factors can independently exert effects and must be excluded
Functional cargo and engineering attributes	Key RNA/protein loading, targeting ligand density, free/encapsulated ratio	Use ddPCR or targeted mass spectrometry for absolute quantification; engineered products must measure ligand density and encapsulation efficiency	Demonstrating only “presence” is insufficient; dose‑response relationships between cargo amount and potency must be established
Formulation and administration attributes	Particle number/protein amount/dose, osmolality, pH, injectability	Report particle number, protein amount, and key cargoes simultaneously; hydrogel products require gelation and release profile determination	Neither protein amount nor particle number alone represents effective dose; multidimensional integration is required
Sterility and biosafety	Microorganisms, endotoxins, abnormal immune activation, and pro‑coagulant risks	Test sterility/mycoplasma/endotoxins per biologic standards; assess TF and phosphatidylserine‑related pro‑coagulant effects	Intra‑articular administration still carries local inflammation risk; allogeneic or platelet‑derived EVs require particular attention
Nonclinical distribution and long‑term safety	Intra‑articular retention, systemic distribution, repeat‑dose toxicity, and tumorigenic risk	Use tracing methods distinguishing intact EVs from free dyes; conduct repeat‑dose and major organ pathology examinations	Pro‑proliferative or gene‑editing EVs require special focus on off‑target and abnormal proliferation risks
Stability and storage	Freeze‑thaw cycles, storage time, aggregation tendency, and potency decay	Establish real‑time/accelerated stability; simultaneously monitor size, markers, key cargoes, and biological potency	Morphological integrity does not guarantee retained activity; functional potency must be the stability release endpoint
Batch‑to‑batch consistency and comparability	Product equivalence after donor/batch/scale changes	Establish CPPs‑CQAs‑potency correlation matrix; conduct functional comparability studies after process changes	With complex compositions, markers alone cannot prove equivalence; the potency matrix is central
Additional attributes of EV‑material combination products	Targeting ligand stability, ligand density, material release profile, and degradation products	Assess uptake differences between target and non‑target cells; hydrogels/microspheres require burst, cumulative release, and mechanical testing	Combination products increase immunogenicity and process complexity; should be evaluated rigorously as combination biologics

### 6.5 Current clinical evidence for EVs in osteoarthritis

To date, evidence for EVs in knee osteoarthritis remains predominantly from cellular and animal studies. A 2026 review noted that preclinical evidence supports the anti-inflammatory, immunomodulatory, and chondroprotective potential of EVs. However, standardized manufacturing, potency assays, and high-quality randomized trials remain insufficient to support routine clinical application (102).

Current clinical studies on osteoarthritis EVs lack uniform standards for yield, dose, and administration regimens. Nominal particle doses in clinical protocols have spanned more than two orders of magnitude. Regimens include single administration, three administrations on days 0/21/42, and repeated administration at 90-day intervals. Published human osteoarthritis studies have already shown some inconsistency in efficacy. A randomized, triple-blind, placebo-controlled trial of placental MSC-EV showed that a single injection was well tolerated. However, at 6 months, clinical scores and MRI parameters did not show superiority over saline control (140). In contrast, a small-sample randomized double-blind dose-escalation study using human umbilical cord MSC exosomes reported improvements in clinical scores and MRI parameters after administration of 3-5 × 10^11 particles. However, because this study mainly compared different doses and did not include a placebo group, it is currently difficult to determine how much of the observed improvement can be clearly attributed to exosome treatment (141). Another clinical-grade UC-MSC-sEV project established a relatively standardized and reproducible production system and reported 12-month safety results from one case/early first-in-human application. However, this should currently be regarded as proof of concept, not a completed efficacy confirmation trial (99). There are also three non-randomized, open-label, multicenter trial protocols using cell-free stem cell-derived extracts rich in EVs (86, 142, 143). The placental, umbilical cord, and other allogeneic MSC-derived EVs used in various clinical studies may differ in cargo and potency. These differences cannot currently be interpreted as evidence of an established optimal dose or optimal treatment frequency. Truly comparable dose-escalation, pharmacokinetic, and potency studies are still very limited. In addition, the endpoints used in different studies vary, including pain, functional scores, MRI cartilage parameters, synovitis, and safety. Therefore, the observed inconsistencies are more likely influenced by product heterogeneity, dose and frequency, patient phenotype and disease stage, small sample sizes, and study design differences. They do not prove that one cell source or one isolation method is naturally superior to others. Thus, there remains a significant gap between the strong anti-inflammatory, chondroprotective, and regenerative effects observed in numerous rodent experiments and the high-quality randomized controlled clinical evidence in human osteoarthritis. Future clinical studies should link key quality attributes with validated potency assays. They should report particle number, protein amount, and functional dose indicators. Study designs should stratify patients based on osteoarthritis phenotype and structural stage. Repeated dosing regimens should be pre-specified. Endpoints for symptom improvement should be distinguished from structural disease modification endpoints. Since short-term pain relief, synovitis suppression, and formation of cartilage repair with long-term mechanical function are biologically different levels of outcomes, longer follow-up is also needed (**Table 3**).

**Table 3:** EVs products in clinical trials for osteoarthritis and their dose strategies and administration regimens.

EV product	Disease/model	Dose	Administration regimen	References
Clinical-grade UC-MSC-sEV	Knee OA, KL II-III	2×10^9, 6×10^9, 2×10^10 particles	Single intra-articular injection	(99)
Placental MSC-EV	Knee OA, KL II-III	5mL, ~7×10^9 particles (~3.5×10^10 particles)	Single intra-articular injection	(140)
hUC-MSC exosomes, MR-13-24-017929	Knee OA, KL II-III	3×10^11, 4×10^11, or 5×10^11 particles	2.5mL each, intra-articular injection on days 0, 21, 42	(141)
Umbilical cord-derived Wharton’s Jelly (contains EVs)	Knee OA, KL II-III	Unknown	Single intra-articular injection	(142)
Cell-free stem cell-derived extract formulation (contains EVs)	Knee OA, KL II-III	2mL	Single intra-articular injection	(143)
Autologous peripheral blood derived circulating EVs (C-EVs)	TMJ OA	20 mL (low dose) or 50 mL (high dose)	Three consecutive intra-articular injections fortnightly	(86)

## 7 Discussion and perspectives

With the rapid development of EVs research, significant differences remain among studies in terminology, isolation methods, and particle characterization. To improve the rigor, reproducibility, and consistency of research in this field, the International Society for Extracellular Vesicles (ISEV) has established the Minimal Information for Studies of Extracellular Vesicles (MISEV) guidelines. These are continuously updated, with MISEV2023 currently serving as the latest consensus guidance (144, 145). According to MISEV2023, EVs are particles released by cells, enclosed by a lipid bilayer, and incapable of self-replication. In contrast, “exosome” specifically refers to EVs of endosomal origin. Therefore, this term should be used cautiously unless their subcellular biogenesis origin is fully substantiated. It is particularly important to note that different EV subtypes overlap considerably in size and molecular composition. Moreover, some proteins traditionally considered “exosome markers” are not exclusive to endosome-derived EVs (146). Similarly, nanoparticle tracking analysis (NTA) provides important information on particle size and concentration. However, NTA alone cannot prove the vesicular nature or endosomal origin of particles. The reproducibility of its results also depends on standardized experimental procedures and reporting standards (147). Therefore, studies that previously defined particles as “exosomes” based solely on particle size, isolation method, or a limited marker panel without adequate EV characterization should be interpreted with caution. This review therefore primarily uses the general term “EV.” The term “exosome” is used only when there is sufficient evidence supporting endosomal origin, or when accurately paraphrasing the terminology used in the original studies.

In addition, matrix vesicles (MVs) need to be distinguished from the small EV/exosome populations that currently dominate therapeutic EV research. Historically defined, MVs are membrane-bound extracellular particles associated with the extracellular matrix of mineralizing tissues. They are particularly closely related to the initiation and regulation of biomineralization. In articular cartilage, MVs can be isolated from both normal and osteoarthritic tissues, and they differ in protein/enzyme composition and mineralization capacity (148). MVs derived from human osteoarthritic cartilage can also generate calcium pyrophosphate dihydrate (CPPD) and apatite-like mineral phases *in vitro*. This suggests they may participate in pathological cartilage calcification (149). In a broad conceptual sense, MVs overlap with EVs because they are also membrane-enclosed particles released by cells into the extracellular environment. However, MVs cannot be automatically equated with exosomes or traditionally defined small EVs. The fundamental reason is that the three are defined on different bases. MVs are historically defined primarily by their localization in the extracellular matrix and their mineralization function. “Exosome” emphasizes their biogenesis from the endosomal system. Small EV (sEV) is more of an operational term based on size and isolation characteristics. Moreover, the isolation methods for MVs may also differ from those for EVs free in synovial fluid or culture medium. This is because a considerable portion of MVs may be embedded in or bound to the extracellular matrix. Therefore, in this review, “matrix vesicle” is used only when discussing extracellular particles related to cartilage mineralization, calcification, or matrix-bound EV signaling. “EV/sEV” is used to describe the broader population of extracellular particles involved in intercellular communication and therapeutic delivery.

Current osteoarthritis EV studies often follow the standard path of “sequencing to screen differential miRNAs – predicting target genes – dual-luciferase validation – mimic and inhibitor rescue.” This strategy helps discover candidate pathways. However, it tends to produce an oversimplified narrative: a particular EV relies on one miRNA acting on one target gene to explain all its anti-inflammatory and regenerative effects. In reality, the copy number of a specific miRNA carried by a single EV may be very low. Only a portion of EVs in the preparation contain that cargo. Proteins, lipids, and other RNAs may simultaneously participate in recipient responses. A decrease in effect after knocking down a certain miRNA only proves its involvement. It does not mean it is the sole active ingredient. Future studies need to adopt methods combining quantitative multi-omics and causal perturbation. First, RNA, protein, lipid, and metabolite profiles of donor cells, total EVs, and functional EV subpopulations should be compared. This will clarify which molecules are selectively loaded. Second, CRISPR, RNA-binding protein knockout, cargo sorting site mutation, and EV subpopulation immunocapture can be used to validate key components. Third, single-cell transcriptomics, phosphoproteomics, metabolic flux, and spatial imaging should be used in recipient cells to observe EV-induced state transitions. This is more informative than measuring only a few endpoints. Ultimately, a multi-layer network model of “preparation components – recipient cell state – tissue outcome” needs to be established. EV effects may also exhibit non-linear and threshold behaviors. Low doses may be insufficient to trigger recipient state transitions. Excessively high doses may cause particle aggregation, phagocyte overload, or non-specific uptake. Different cargo may synergize with each other. For example, one miRNA lowers the inflammatory threshold, a EV protein simultaneously restores mitochondrial function, and lipid components alter membrane receptor signaling. Systems biology analysis can help identify combination features that determine efficacy and provide a basis for establishing multi-parameter release standards.

One of the key unresolved questions in EV research is why cells select certain cargo for release under specific pathological states. A study on FMRP-mediated entry of miR-155-5p into EVs from inflammatory macrophages provides a model. The study not only found elevated miRNA in EVs but also demonstrated the involvement of RNA-binding proteins in selective sorting. It linked this process to disease progression and engineered therapy (150). Future studies should further resolve how ESCRT, Rab proteins, tetraspanins, lipid composition, and RNA modifications determine EV subpopulations and cargo in osteoarthritis. A second question is whether bioactive cargo can enter the cytoplasm or other functionally relevant intracellular compartments before being completely degraded in lysosomes. Cellular uptake does not automatically equate to functional delivery of EV cargo. After EVs enter cells via endocytosis, a considerable portion of internalized particles are transported to late endosomes and lysosomes. Their membrane structures and carried cargo may then be degraded or enter intracellular recycling. Most *in vitro* studies assume that EVs will be taken up by all cells. However, in the joint *in vivo*, uptake efficiency differs among synovial macrophages, chondrocytes, fibroblasts, endothelial cells, and bone cells. Several possible mechanisms have been proposed or experimentally validated. In some cell systems, EVs can fuse directly with the plasma membrane. After endocytosis, the EV membrane may also undergo back-fusion with the endosomal limiting membrane. This releases the RNA, protein, and other cargo from the EV lumen into the cytoplasm. Joshi et al. directly observed interactions between EVs and endosomes. They found that EV cargo could be released from acidified endosomal compartments. This process depended on endosomal acidification (151). Studies on functional nucleic acid delivery by erythrocyte-derived EVs similarly suggest that cargo can escape from late endosome/lysosome-related compartments and exert functions (152). However, one cannot assume that all EVs can efficiently achieve endosomal escape. Under different EV subpopulations, recipient cell types, endocytic pathways, and experimental conditions, a large portion of EVs may remain trapped in endosomes or eventually be degraded in lysosomes. In osteoarthritis research, cargo release reporter systems, endosomal co-localization analysis, membrane fusion experiments, and functional rescue assays should be combined as much as possible. This will distinguish between “particle localization,” “EV internalization,” “endosomal escape,” and “cytoplasmic functional cargo delivery.” The efficiency of endosomal escape of EVs in articular chondrocytes and synovial macrophages remains an important unresolved issue determining their actual pharmacodynamic effect. A third question is whether lipid components in EVs can directly restore cartilage homeostasis or promote cartilage regeneration. Lipid molecules such as cholesterol and sphingomyelin in EVs can affect membrane curvature, surface charge, cellular uptake, and membrane fusion. CD63 has been shown to regulate cholesterol sorting into intraluminal EVs and further influence exosome-related cholesterol transport (153). In equine synovial fluid EVs, osteoarthritis was associated with altered fatty acid profiles (154). Further phospholipidomics and proteomics integration analysis also found that relative levels of EV-associated phospholipid classes, including sphingomyelin, phosphatidylcholine, phosphatidylinositol, and phosphatidylserine, could change with OA pathological states (155). These results suggest that EV lipids may serve both as disease markers and as participants in regulating EV-cell interactions. Future studies should combine quantitative lipidomics with specific lipid depletion, supplementation, or reconstitution experiments. This will determine whether specific EV lipids can directly regulate chondrocyte inflammatory signaling, membrane fusion, mitochondrial metabolism, or macrophage responses independently of RNA and protein cargo.

Disease severity likely determines important therapeutic windows for EV therapy. The OARSI grade mainly reflects the depth and structural severity of cartilage degeneration. As the OARSI grade increases, the mechanical properties of cartilage gradually deteriorate. Studies on human articular cartilage have shown a stepwise decrease in cartilage dynamic modulus from OARSI grade 0 to 4. Tissue permeability increases with degeneration severity (156, 157). These structural changes may have dual or even opposing effects on EV therapy. At relatively early histological grades, the cartilage surface is still relatively intact and retains more viable chondrocytes. Therefore, EVs that can inhibit synovial inflammation, restore mitochondrial/autophagy homeostasis, or rebalance matrix metabolism may have a greater opportunity to maintain and rescue endogenous tissue. At the mid-stage, with increasing chondrocyte senescence, subchondral bone remodeling, synovitis, and matrix structural disorganization, multi-target or sequential EV treatment strategies may be needed. At the late erosion stage, viable chondrocytes and load-bearing extracellular matrix are largely lost. Even if EVs can still exert anti-inflammatory or analgesic effects, the ability of cell-free EV preparations alone to achieve structural regeneration may be significantly limited. However, direct evidence comparing EV efficacy by pre-treatment OARSI histopathological grade is currently limited. Current knee osteoarthritis EV clinical studies mostly use Kellgren-Lawrence (KL) radiographic grading. For example, the placental MSC-EV randomized trial and the recent hUC-MSC exosome study mainly included KL II-III patients. Therefore, efficacy in severe end-stage osteoarthritis is still insufficiently evaluated (140, 141). Therefore, future preclinical studies, where conditions permit, should report baseline OARSI grade before or at treatment initiation. They should further analyze the interaction between treatment effect and disease severity. Clinical trials should perform prospective patient stratification based on radiographic severity, cartilage integrity, synovitis status, and other molecular or imaging endophenotypes.

Most current osteoarthritis EV studies use DMM, ACLT, collagenase, or MIA-induced rodent models. Different models favor mechanical instability, inflammatory destruction, or chemical chondrocyte injury. They are not interchangeable. The destabilization of the medial meniscus (DMM) model can produce relatively slowly progressing mechanical instability. It is closer to mechanical load-driven or post-traumatic early-to-mid osteoarthritis. However, the commonly used young animal DMM model cannot simulate the advanced age, metabolic abnormalities, and multimorbidity background present in most clinical patients. The anterior cruciate ligament transection (ACLT) model, with or without meniscal injury, causes more sudden and severe joint instability. It is suitable for post-traumatic osteoarthritis research. However, it may overrepresent acute mechanical injury and drive structural lesions faster than the slowly progressing primary osteoarthritis commonly seen clinically. The collagenase-induced osteoarthritis model simultaneously features ligament laxity and pronounced synovial inflammation. Therefore, it can be used to simulate the “mechanical instability-inflammation dominant” phenotype. However, enzyme-mediated tissue damage and susceptibility differences between animal strains and sexes limit its direct clinical extrapolation. The monosodium iodoacetate (MIA) model causes rapid chondrocyte metabolic damage, cartilage destruction, and stable pain behaviors. It is therefore commonly used for pain mechanism and proof-of-concept pharmacodynamic studies. However, its chemical injury mechanism and rapid course differ considerably from the natural evolution of human chronic osteoarthritis. Transcriptomic comparisons also show relatively low molecular concordance between MIA model cartilage and human osteoarthritic cartilage (158, 159). In contrast, spontaneous and age-related osteoarthritis models are better suited for simulating gradually accumulating cellular senescence, chronic matrix degeneration, and age-dependent joint remodeling. Their drawbacks are long disease course and large individual variability. High-fat diet/obesity models are more suitable for metabolic-inflammatory osteoarthritis. However, systemic metabolic abnormalities and increased mechanical load due to weight gain often co-exist and are difficult to completely separate. It is worth noting that the effects of the same molecular target are not necessarily consistent across different osteoarthritis phenotypes. A recent update analysis of genetically modified mouse studies found that about one-third of the molecular targets examined differed in direction or magnitude of effect between post-traumatic OA models and spontaneous/age-related OA models. This further emphasizes that preclinical studies must pay attention to matching models with disease endophenotypes (160). This issue is particularly important for therapeutic EVs. Their biological effects are highly dependent on recipient cell state, inflammatory environment, metabolic background, age, and tissue accessibility. Current osteoarthritis EV research still relies heavily on young, normal-weight laboratory rodents. Systematic inclusion of aged animals, female animals, obese/metabolically abnormal animals, and multimorbidity models remains significantly insufficient. Age and sex are not minor experimental variables. A scoping review of large animal osteoarthritis studies identified 9,727 publications. However, only 238 studies reported sufficient model, sex, and skeletal maturity information for analysis. Among these, only 26 studies formally evaluated the effects of sex and/or age (161). Therefore, future therapeutic EV studies should not rely on a single animal model to prove efficacy. They should establish a “model combination validation strategy.” Multi-factor models that simultaneously incorporate aging, obesity, diabetes, or other clinically relevant comorbidities should be established. This will verify whether EVs remain effective in the adverse systemic environments that real patients face. Before entering clinical trials aimed at efficacy validation, promising EV preparations should also be validated in large animals. The goal of large animal studies should not be limited to again demonstrating histological improvement. They should systematically determine cross-species dose conversion, cartilage penetration and intra-articular retention, local tolerance to repeated administration, proportion entering systemic circulation, gait and weight-bearing changes, MRI structural outcomes, and long-term mechanical function.

Osteoarthritis patients may respectively present with synovitis-dominant, subchondral bone remodeling-dominant, metabolic inflammation-dominant, post-traumatic mechanical instability, senescent cell accumulation, or pain sensitization phenotypes (162). The same EV preparation is unlikely to produce consistent effects across all phenotypes. Synovitis-type patients may be more suitable for macrophage-targeting and inflammasome-inhibiting EVs. Senescence-type patients may need senolytic or rejuvenating EVs. Subchondral bone-type patients may require EVs that regulate osteocyte EVs and vascular-osteogenic coupling. Patients with large cartilage defects may need EVs combined with scaffolds, cells, or surgical repair. Synovial fluid and blood EVs themselves can be used for patient stratification. EV surface proteins and cargo can reflect source cell states. They may be more stable than single free cytokines (45). The sex-related synovial fluid EV miRNA differences found by Kolhe et al., the non-coding RNA networks in synovial fluid EVs, FMRP associated with disease progression in plasma EVs, and the C1QBP-positive EVs in the temporomandibular joint osteoarthritis study all suggest that EVs can serve as companion diagnostics and efficacy monitoring tools (44, 86). However, blood EV sources are complex. Local joint signals may be diluted by EVs from other organs. Synovial fluid collection is somewhat invasive. Future studies could use tissue source markers, multi-parameter single-particle detection, and machine learning to construct combined features. This is preferable to relying on a single miRNA (163). Precision therapy also requires defining the timing of administration. Using potent immunosuppressive EVs during an acute inflammatory flare may hinder necessary debris clearance. When inflammation persists long-term, immune reset must first be accomplished before regenerative EVs can take effect. Thus, a staged regimen could be considered. First, macrophage or synovium-targeted EVs are used to reduce pathological inflammation and clear senescent cells. Then, cartilage-targeted, matrix-synthesizing, and mitochondrial-repairing EVs are used to promote regeneration. Finally, material and mechanical interventions are used to maintain tissue maturation (164). Compared with concentrating all functions into a single ultra-complex preparation, sequential therapy may better match the pathological process of osteoarthritis. It also facilitates separate control of quality and risk for each product.

## 8 Conclusion

EVs provide a communication-based perspective on osteoarthritis that transcends “cartilage wear and tear”. Pathological EVs released by inflammatory synovial cells, stressed chondrocytes, osteocytes, and vascular cells propagate inflammatory, senescence, mitochondrial damage, and catabolic signals among joint tissues, forming a self-sustaining pathological EV cycle. Therapeutic EVs can remodel the joint environment from sustained damage toward permissive regeneration by reshaping macrophage status, inhibiting inflammasomes, restoring autophagy and mitochondrial homeostasis, attenuating programmed cell death, clearing senescent cells, and promoting matrix synthesis. Their core value lies not in replacing any single cytokine, but in intervening simultaneously in immunity, metabolism, and tissue repair as multi-component information packets. However, the dual nature of EVs also means that any therapeutic effect depends on source, cargo, recipient, and disease stage. Future research must move from single-miRNA narratives to multi-component causal mechanisms, from generic MSC EVs to phenotype-matched and temporally sequenced therapy, and establish manufacturing, potency, and safety evaluation systems aligned with MISEV principles and regulatory requirements. Only when EVs are shown to stably disrupt pathological communication, accomplish immune resetting, and achieve osteochondral repair with long-term mechanical function can they truly transform from experimental nanocarriers into disease-modifying biologics for osteoarthritis.
